# Genome-Wide Identification and Immune Response Analysis of Serine Protease Inhibitor Genes in the Silkworm, *Bombyx mori*

**DOI:** 10.1371/journal.pone.0031168

**Published:** 2012-02-13

**Authors:** Ping Zhao, Zhaoming Dong, Jun Duan, Genhong Wang, Lingyan Wang, Youshan Li, Zhonghuai Xiang, Qingyou Xia

**Affiliations:** 1 State Key Laboratory of Silkworm Genome Biology, Southwest University, Chongqing, China; 2 Institute of Agricultural and Life Sciences, Chongqing University, Chongqing, China; New Mexico State University, United States of America

## Abstract

In most insect species, a variety of serine protease inhibitors (SPIs) have been found in multiple tissues, including integument, gonad, salivary gland, and hemolymph, and are required for preventing unwanted proteolysis. These SPIs belong to different families and have distinct inhibitory mechanisms. Herein, we predicted and characterized potential SPI genes based on the genome sequences of silkworm, *Bombyx mori*. As a result, a total of eighty SPI genes were identified in *B. mori*. These SPI genes contain 10 kinds of SPI domains, including serpin, Kunitz_BPTI, Kazal, TIL, amfpi, Bowman-Birk, Antistasin, WAP, Pacifastin, and alpha-macroglobulin. Sixty-three SPIs contain single SPI domain while the others have at least two inhibitor units. Some SPIs also contain non-inhibitor domains for protein-protein interactions, including EGF, ADAM_spacer, spondin_N, reeler, TSP_1 and other modules. Microarray analysis showed that fourteen SPI genes from lineage-specific TIL family and Group F of serpin family had enriched expression in the silk gland. The roles of SPIs in resisting pathogens were investigated in silkworms when they were infected by four pathogens. Microarray and qRT-PCR experiments revealed obvious up-regulation of 8, 4, 3 and 3 SPI genes after infection with *Escherichia coli*, *Bacillus bombysepticus*, *Beauveria bassiana* or *B. mori* nuclear polyhedrosis virus (BmNPV), respectively. On the contrary, 4, 11, 7 and 9 SPI genes were down-regulated after infection with *E. coli*, *B. bombysepticus*, *B. bassiana* or BmNPV, respectively. These results suggested that these SPI genes may be involved in resistance to pathogenic microorganisms. These findings may provide valuable information for further clarifying the roles of SPIs in the development, immune defence, and efficient synthesis of silk gland protein.

## Introduction

Serine proteases (SPs) constitute nearly one-third of all the known proteolytic enzymes and modulate the bioactivity of target proteins in a timely manner through proteolytic cleavage [1]. When these serine proteases are no longer in need, they will be inactivated by serine protease inhibitors (SPIs). Many SPIs from insects have been purified from the integument, genital tract, salivary gland, and hemolymph. SPIs not only play key roles in insect digestion, metamorphosis and development, but also are important components of immune system [2], [3], [4], [5], [6], [7], [8], [9]. SPIs are named as trypsin inhibitors (TIs), chymotrypsin inhibitors (CIs), elastase inhibitors (EIs) and subtilisin inhibitors (SIs) according to their different inhibitory target proteases. SPIs also can be classified into three types, namely, canonical inhibitors, non-canonical inhibitors, and serpins based on the mechanism of action [10]. Canonical inhibitors are usually small proteins (14∼200 amino acid residues) and can bind to protease through an exposed convex binding loop [11]. According to sequence homology, position of active center, and disulfide bond structure, the canonical inhibitors can be divided into at least 20 families [10], [12]: Bowman-Birk family, Kazal family, BPTI-Kunitz family, etc. Non-canonical inhibitors only present in blood-sucking insects and leeches. Serpins are significantly large proteins (of about 40∼50 kDa). Reactive centre loop (RCL) of serpin is located in the C-terminal of peptide chain which acts as a “bait” for SP [12], [13]. The cleavage of RCL results in a profound conformational change within the serpin. Therefore serpin is known as a suicide inhibitor [12], [13]. Currently, protease inhibitors are classified into 88 families on the basis of amino acid homology, and are listed in the MEROPS database (http://merops.sanger.ac.uk/cgi-bin/family_index?type=I).

Various insect SPIs have been purified, and their inhibitory specificities were then analyzed. Kang et al. (1980) purified a protein from *Drosophila melanogaster* which inhibits bovine alpha-chymotrypsin activity but did not exhibit any inhibitory activity against trypsin [14]. Kanost et al. (1990) isolated four serpins from hemolymph of fifth instar larvae of *Manduca sexta*, two of which are specific to chymotrypsin, one is specific to elastase and one is to trypsin [15]. Ramesh et al. (1988) purified two Kunitz-type inhibitors from the hemolymph of *M. sexta* and found that they could inhibit serine proteases including trypsin, chymotrypsin, and plasmin [16]. Boigegrain et al. (1992) isolated two protease inhibitors from the plasma of *Locusta migratoria*, which showed a strong inhibiting activity toward alpha-chymotrypsin, elastase and phenoloxidase [17]. Hamdaoui et al. (1998) purified five SPIs from ovaries of *Schistocerca gregaria* and designated them SGPI-1∼5, which all showed an in vitro inhibiting activity towards alpha-chymotrypsin [6].

Researches on SPIs in *Bombyx mori* are relatively intensive because *B. mori* is the lepidopteran model insect and has important economic value. In 1960, Morita et al. (2005) first purified protease inhibitors from larval hemolmph of silkworm [18]. At least 16 *B.mori* hemolymph SPIs from kunitz family and serpin family show inhibitory activity to chymotrypsin and are thus named as CI-1∼CI-13. Fujii et al. (1996) found that these CIs are controlled by 5 genes (Ict-A, B, D, E and H) and have abundant polymorphism in different geography strains of silkworm [19], [20]. Six high-content CIs were purified and their animo acid sequences and physicochemical properties have been analyzed [21], [22], [23], [24], [25], [26], [27]. CI-13 of kunitz family exists in the active ingredient of phenoloxidase precursor activation [28]. CI-b1 of kunitz family binds to LPS and scavenge intruding bacteria through interacting with lipopolysaccharides (LPS) [29]. CI-8 of serpin family has receptor protein in the midgut and shows inhibitory activity to protease in the digestive juice [24]. The studies on trypsin inhibitors (TIs) are relatively fewer than those on CIs of the silkworm. Six cocoon shell-associated TIs (CSTIs-I, II, III, IV, V and VI) in cocoon proteins showed polymorphism distribution among 64 silkworm strains [30]. These CSTIs displayed different distributions in cocoons: outer layer of cocoon contains no CSTI-I, whereas inner layer of cocoon contains all the 6 CSTIs [31]. Further research showed that Kunitz-type CSTI-VI distributes at the anterior and middle silk gland, and can prevent fibroin light chain (Fib-L) from being degraded by proteases [32]. *B.mori* fungal protease inhibitor-F (FPI-F) belongs to a novel serine protease inhibitor family, which was firstly named as *Bombyx* family and recently renamed as trypsin inhibitor like cysteine rich domain (TIL) family [33]. FPI-F was found in the hemolymph and integument of the silkworm larvae and includes eight cysteines forming four disulfide bonds [34]. FPI-F could inhibit subtilisin and fungal proteases from *Aspergillus melleus* and *Beauveria bassiana*, and prohibit germination of conidia and germ tube development of *B.bassiana* [35].

Previous studies showed that insects contain different families of SPIs, which play important roles in the physiological and pathological processes. Although serpin genes have been identified and characterized in many insects such as *Anopheles gambiae*, *Aedes aegypti*, *Manduca sexta*, *Bombyx mori*, *Apis mellifera* and 12 Drosophilid species [8], [36], [37], [38], [39], [40], [41], [42], [43], [44], [45], studies on SPIs from other families still on the biochemical level. In order to reveal more unknown SPIs, this article systematically analyzed the classifications, domain organization, expression characters and immune response of SPIs on a genome-wide scale in the silkworm.

## Results

### Identification and chromosomal distribution of silkworm SPI genes

SPI genes from *D. melanogaster* and other insects were used to blast against the silkworm genome. A total of eighty *B. mori* SPI genes were identified and named as *BmSPI1*∼*BmSPI80* (Table 1 and Table S1), 41 of which had previously been submitted to GenBank, including 34 serpins, 3 Kazal-type, 2 Kunitz-type, 1 WAP-type and 1 amfpi-type genes. Among new identified 39 SPI genes, 25 genes have expressed sequence tag (EST) expression evidence (Table S1). Their nucleotide sequences have been corrected by assembling overlapping EST sequences (Figure S1). However, three SPIs were not identified under our search conditions, namely FPI-F (GenBank Accession No. AAB46908) and two Kunitz-type CIs (GenBank Accession No. BAB83366 and AAW30167). The possible reason may be that these three genes are diversified in sequence among strains because of evolution.

**Table 1:** The SPI domains in the silkworm.

Name	SPI domain	Numbers of SPI domain
BmSPI1∼34	Serpin	1
BmSPI35∼42	TIL	1
BmSPI43	TIL	3
BmSPI44	TIL	1
BmSPI45	TIL	3
BmSPI46	TIL	4
BmSPI47	TIL	6
BmSPI48	TIL	4
BmSPI49	TIL	5
BmSPI50∼55	Kunitz_BPTI	1
BmSPI56	Kunitz_BPTI/Antistasin/WAP	1/3/2
BmSPI57	Kunitz_BPTI/WAP	4/1
BmSPI58	Kunitz_BPTI	8
BmSPI59∼64	Kazal	1
BmSPI65	Kazal	3
BmSPI66	Kazal	1
BmSPI67	Kazal	7
BmSPI68	Kazal	9
BmSPI69	amfpi	1
BmSPI70	Kunitz_BPTI	-
BmSPI71	Pacifastin	2
BmSPI72	Pacifastin	4
BmSPI73	Pacifastin	13/11
BmSPI74	Bowman-Birk	11
BmSPI75∼78	α-macroglobulin	1
BmSPI79	WAP	1
BmSPI80	WAP	2

**Figure 1:**
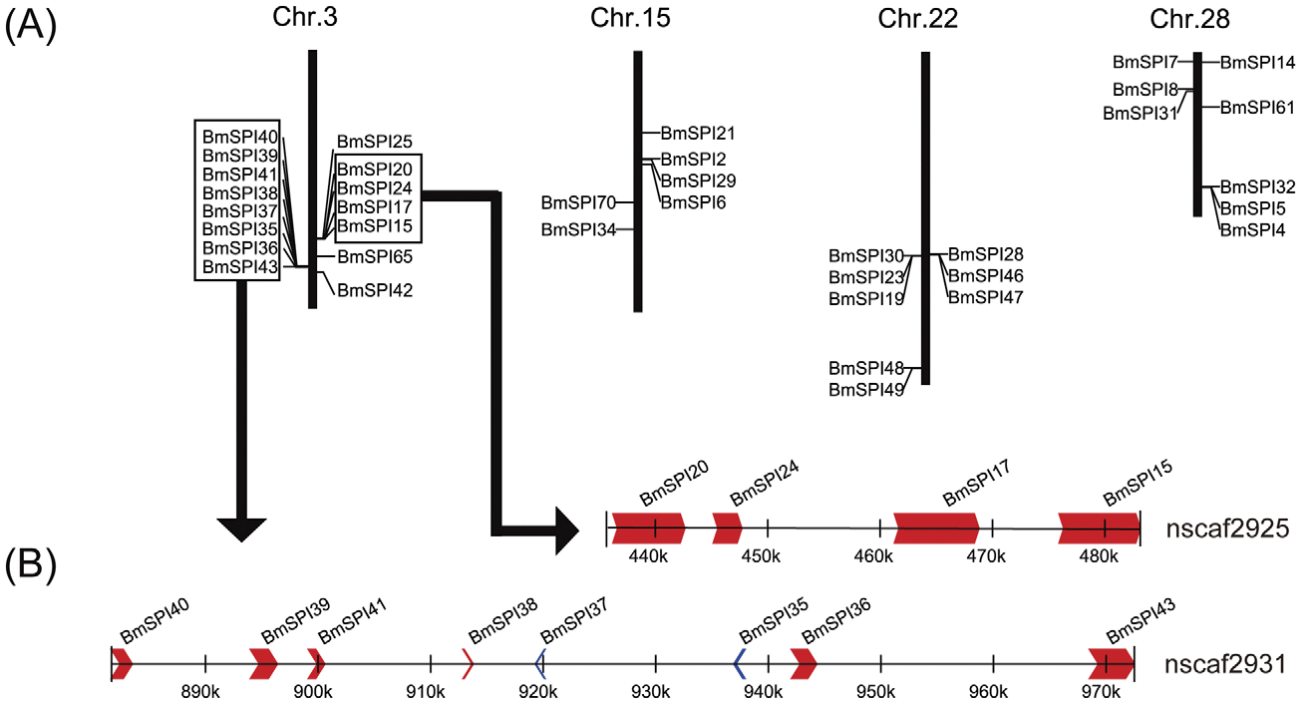
Distribution of SPI genes on silkworm chromosomes. (A) The four Chromosomes with the most number of SPI genes. Many SPI genes are in tandem clusters. (B) An example of SPI genes clustered on chromosome 3. Four genes on nscaf2925 are distributed in a cluster within 50 kb and eight genes on nscaf2931 are distributed in a cluster within 100 kb. The arrowhead indicates the transcriptional orientation of genes.

Seventy SPI genes (89.5%) were successfully localized on silkworm chromosomes, with an uneven distribution (Table S1 and Figure S2). For example, eleven chromosomes have no SPI genes, while chromosomes 3, 15, 22 and 28 have 15, 6, 8 and 8 SPI genes, respectively, accounting for 46.25% SPI genes (Figure 1 and Figure S2). Additional analysis showed that SPI genes exhibit a tandem repeat distribution on chromosomes. For example, there are two tandem repeat gene groups on chromosome 3 (Figure 1), the first group contains *BmSPI15*, *BmSPI17*, *BmSPI20* and *BmSPI24* distributed in a range of 50 kb, and the second includes *BmSPI35∼41* and *BmSPI43* distributed in a range of 100 kb. SPI genes from one tandem repeat gene group are highly similar, suggesting that these genes might derive from gene duplication.

### The inhibitor domains of silkworm SPI genes

In total, 10 SPI domains were identified: TIL [46], Kunitz_BPTI [46], Kazal [46], Antistasin [46], Pacifastin [47], whey acidic protein (WAP) [48], [49], amfpi [50], Bowman-Birk [46], [51], serpin [45] and alpha-macroglobulin [52] (Table 1 and Table S1). These 10 SPI domains can be divided into three types: serpin, alpha-macroglobulin and canoniacal SPIs,.

Thirty-four *B.mori* serpins (*BmSPI1∼34*) have been identified and their reactive sites have been predicted previously [45]. However, *B.mori* alpha-macroglobulins are firstly identified in this study with four members (BmSPI75∼78). In vertebrates, alpha-macroglobulins have been found with inhibition to an large variety of proteases (not only serine proteases, but also cysteine-, aspartic- and metalloproteases) [53]. To control protease activities, alpha-macroglobulins inhibit protease activities by forming macromolecular cages inside which proteases are cross-linked and trapped, rather than blocking the active sites of proteases [54].

The other eight types of SPIs belong to canonical SPIs and thus have canonical inhibition mechanism, which is different from that of serpin and alpha-macroglobulin. Canonical SPIs are rich in cysteine residues for forming disulfide bonds (Figure S3). Multiple sequence alignments revealed that the number and position of cysteine residues are highly conserved in the same family (Figure S3). For example, each of Kunitz_BPTI, Kazal, Antistasin and Pacifastin contains three pairs of intradisulfide bonds, while four pairs exist in WAP, four or five pairs in TIL, and six pairs in amfpi (Figure S3).

We have predicted proteolytic cleavage sites of silkworm canonical SPIs on the basis of the sequence alignments with reported SPIs (Figure S3). The P1 residue locates in amino-termini of the cleavage site, and determines the specific inhibitory activity of SPIs. BmSPI50 (Kunitz family) may inhibit chymotrypsin-like enzymes (with Phe located at the predicted P1 position); BmSPI35, 38, 39, 40 (TIL family), BmSPI52 (Kunitz family), BmSPI66 (Kazal family) and BmSPI79 (WAP family) may be anticipated to inhibit elastase-like SPs (with Ala, Val or Gly located at P1 site); BmSPI44 (TIL family), BmSPI51, 52, 54, 55 (Kunitz family), BmSPI60, 61,64 (Kazal family) and BmSPI69 (amfpi family) probably have inhibitory activity to trypsin-like enzymes (Lys or Arg located at P1 site) (Figure S3). The amino acid sequence of BmSPI69 is similar to that of a small cationic peptide CP8 in the hemolymph of *Maduca sexta* and of a fungal protease inhibitor-1 (AmFPI-1) of the Indian tasar silkworm *Antheraes mylitta* [50]. AmFPI-1 has inhibitory activity against some fungal serine proteases and trypsin, but *Manduca* CP8 did not inhibit the activity of serine proteases [55]. However, it's not certain whether BmSPI69 has inhibitory activity against serine proteases. As for TIL-type Proteins, BmFPI-F, BmSPI36 and BmSPI37, Thr occupies the P1 position. Previous research reported that FPI-F has inhibitory activity to subtilisin and some fungal serine proteases [56], so we speculated BmSPI36 and BmSPI37 may have similar inhibitory activity.

24 of canonical SPIs are single-domain inhibitors while other canonical SPIs contain two or more inhibitor domains (Table 1). SPIs with multiple inhibitor domains also have various P1 positions, so they are likely to have different inhibitory activities (Figure S3) [34], [47], [49], [50], [56], [57], [58]. For instance, BmSPI48 contains four TIL domains, and may have inhibitory activity to both chymotrypsin-like enzymes (with Leu and Phe located at P1 site of the first, third and the fourth domain) and elastase-like enzymes (with Gly located at P1 site of the second domain). BmSPI65 is composed of three Kazal domains, and probably have inhibitory activity to both trypsin-like enzymes (with Lys located at P1 site of the first and third domain) and chymotrypsin-like enzymes (with Tyr located at P1 site of the second domain). Zheng (2007) et al. found BmSPI65 also has inhibitory activity to subtilisin A [59].

### The non- inhibitor domains of silkworm SPI genes

**Figure 2:**
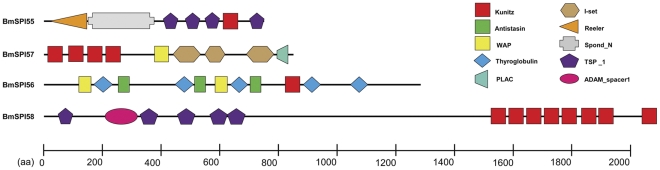
Domain organization of some SPIs in Kunitz_BPTI family in *Bombyx mori*. Rectangular boxes in different colors (red, green and yellow) represent the serine protease domain. The rhombic box in blue represents the cysteine protease domain. Boxes in other shapes and colors represent the non-inhibitor domains. The sizes (length of amino acid residues) of domains are marked by the scale.

This study also identified SPIs with both inhibitor and non-inhibitor domains. Multiple domains in one protein may act synergistically in the processes of tissue morphogenesis. BmSPI74 contains 11 Bowman-Birk domains at least and also many EGF (epidermal growth factor) and YLP domains, and terminates in a zona pellucida domain. BmSPI74 is similar to Dumpy in *Drosophila*, which is a gigantic 2.5 MDa extracellular matrix protein and was predicted to be a membrane-anchored fibre of almost a micrometer in length [60]. Previous studies explained Dumpy's contribution to tissue morphogenesis through its regulation of mechanical properties at epidermal-cuticle attachment sites [60]. However, the function of Bowman-Birk domain in this protein is still unclear. BmSPI55∼58 from Kunitz family contain other non-inhibitor domains (Figure 2). BmSPI57 (Figure 2) has various types of domains, including Kunitz_BPTI, immunoglobulin-like, WAP and PLAC domains. BmSPI57 is homologous to lacunin, which is a large extracellular matrix protein and accompanies morphogenesis of epithelial monolayers in *M.sexta* [61]. BmSPI55 (Figure 2) is structurally composed of one Kunitz_BPTI domain, one spond_N domain, one reeling domain and four thrombospondin domains. BmSPI55 is homologous to F-spondin, which is also an extracellular matrix protein and may be involve in the growth and guidance of axons in nervous system of vertebrate [62]. BmSPI58 (Figure 2) contains eight Kunitz_BPTI domains, five thrombospondin_1 domains and one ADAM_spacer1 domain. BmSPI58 is similar to papilin, which interacts with several extracellular matrix components and ADAMTS enzymes [63]. Papilins are essential to embryonic development of *D.melanogaster*. BmSPI70 was predicted as a homologue of human Inter-alpha-trypsin inhibitor (ITI) and arranged into the Kunitz family. The human ITI consists of one light chain (two tandem Kunitz domain) and a variable set of heavy chains. ITI heavy chains were reportedly could stabilize the cumulus extracellular matrix by binding hyaluronan and involved in the development of embryo and liver [64], [65], [66]. This study has only identified the corresponding heavy chain of BmSPI70.

### Spatial expression profiles of silkworm SPI genes

**Figure 3:**
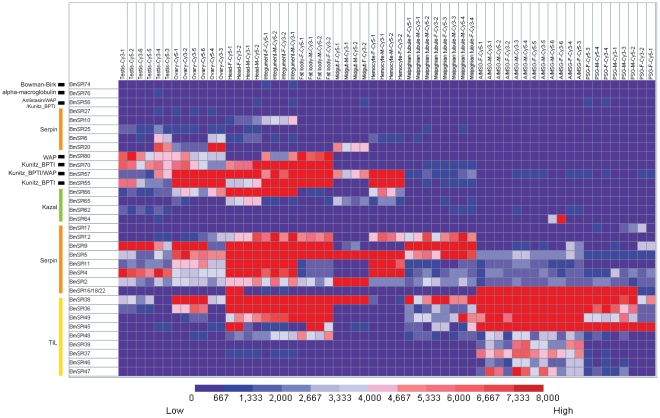
Tissue expression profiles of silkworm SPI genes. Gene expression levels are represented by red (higher expression) and blue (lower expression) boxes. The columns represent ten different tissue or organ samples: testis, ovary, head, integument, fat body, midgut, hemocyte, malpighian tubule, A/MSG (anterior/median silk gland) and PSG (posterior silk gland). The column name is composed as “sample name-labeling dye- experiment order”.

**Figure 4:**
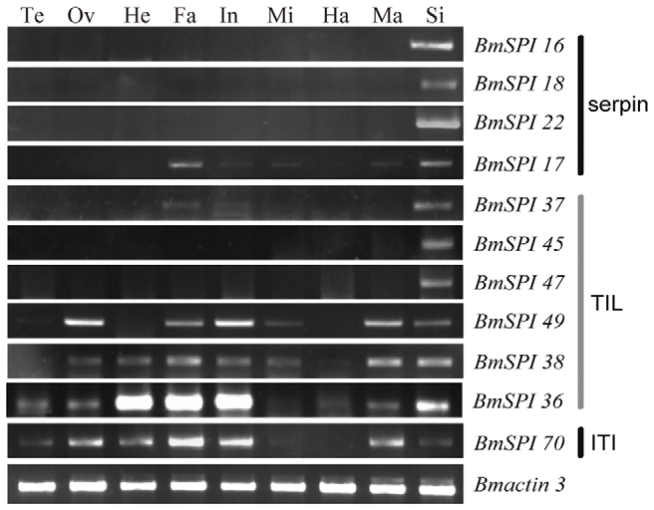
Expression patterns of some silkworm SPI genes showing tissue-specificy in silkworm larvae on day 3 of the fifth instar. Semi-quantitative RT-PCR amplification of total RNA with BmSPI-specific oligonucleotides. Te, testis; Ov, ovary; He, head; Fa, fat body; In, integument; Mi, midgut; Ha, haemocyte; Ma, Malpighian tubules; Si, silk gland. The silkworm cytoplasmic actin A3 gene (Bmactin3, GenBank accession No. U49854) was used as internal control, and denoted by *Bmactin3*.

Microarray analysis showed that 35 SPI genes have transcriptional activity in at least one tissue/organ of day-3 fifth instar larvae according to a stringent criterion for definition of expressed genes (Table S2 and Figure 3). Silk gland had the highest number (19) of expressed genes among the ten tissues or organs, while the number of expressed genes in other tissues or organs is head-18, testis-16, ovary-16, integument-16, fat body-15, malpighian tubule-13, hemocytes-11 and midgut-7 (Figure 3). SPIs have opposite tissue distributions tendency when compared with SPs. The highest number of SPs distribute in the midgut but lowest number of SPs is in the silk gland [67]. Various SPIs expressed in the silk gland may be involved in the silk synthesis and secretion. Further analysis revealed that SPIs in the silk gland belong to the silkworm-specific families. Nine TIL-type SPI genes (*BmSPI36∼39* and *BmSPI45∼49*) and four group F members in serpin family (*BmSPI16, 17, 18* and *22*) were expressed in the silk gland highly or exclusively. The results of semi-quantitative RT-PCR confirmed that *BmSPI16*, *18*, *22*, *45* and *47* were expressed in the silk gland exclusively, but *BmSPI36*, *38* and *49* were expressed in the silk gland, fat body and integument, etc (Figure 4). These results were consistent with the microarray data. Exclusive expression patterns in other tissues were also revealed by microarray analysis. For example, exclusive expression was seen for *BmSPI10* (serpin family) in the head and integument, *BmSPI25* (serpin family) and *BmSPI62* (Kazal family) in the testis.

### Temporal expression profiles of silkworm SPI genes

**Figure 5:**
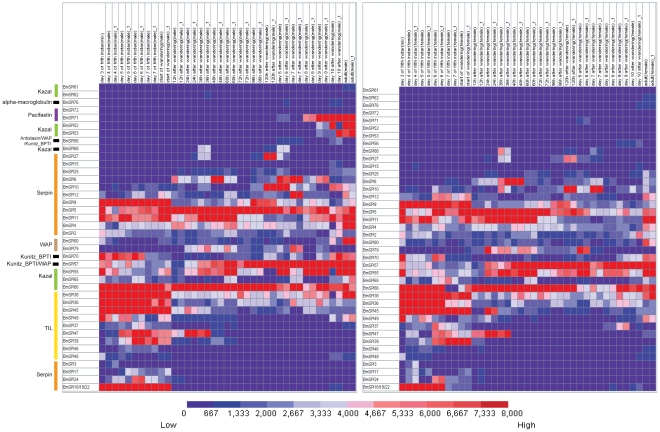
Expression profiles of SPI genes during different developmental stages. Gene expression levels are represented by red (higher) and blue (lower) boxes. The columns represents twenty different sample time points: day 3, 4, 5, 6, 7 of the fifth instar, start of wandering, 12th, 24th, 36th, 48th, 60th, 72nd, 96th, 120th hour after wandering; day6, day7, day8, day9, day10 after wandering and adult. Word “mix” in the column name represents that the sample came from both gender; word “m” in the column name represents that the sample came from male silkworm; word “f” in the column name represents that the sample came from female silkworm.

Using microarray analysis, we found that fourteen silkworm SPI genes are highly expressed during metamorphosis from the fifth instar larva to moth (Table S3 and Figure 5). Exclusive temporal expression patterns were also observed. For example, nine SPI genes from serpin and TIL families were exclusively expressed at the fifth instar of larval stage. Since most of these 9 genes showed high transcript level in the silk gland (Figure 3) and no expression at the pupae and moth stage, it was possibly caused by apoptosis of silk gland cell at the pre-pupae stage. Two SPI genes (BmSPI68 in Kazal family, BmSPI27 in serpin family) were exclusively expressed at the 36^th^ hour, the 5^th^ day and 6^th^ day after wandering (Figure 5). This is consistent with that of a serine protease gene *BmSP116* in *B.mori* [67]. The majority of SPI genes were expressed in both male and female silkworms. However, three SPI genes (BmSPI71 in Pasifastin family, BmSPI52 and 53 in Kazal family) showed sex-biased expression during the metamorphosis, highly and exclusively expressed in males, while minimally in females (Figure 5). Their expression pattern is extremely similar to that of fifteen serine protease genes previously found in *B.mori* [67]. Therefore, the three SPIs may be involved in the activity regulation of serine proteases in male silkworm moth.

### Expression changes of SPI genes after infection with pathogen

**Figure 6:**
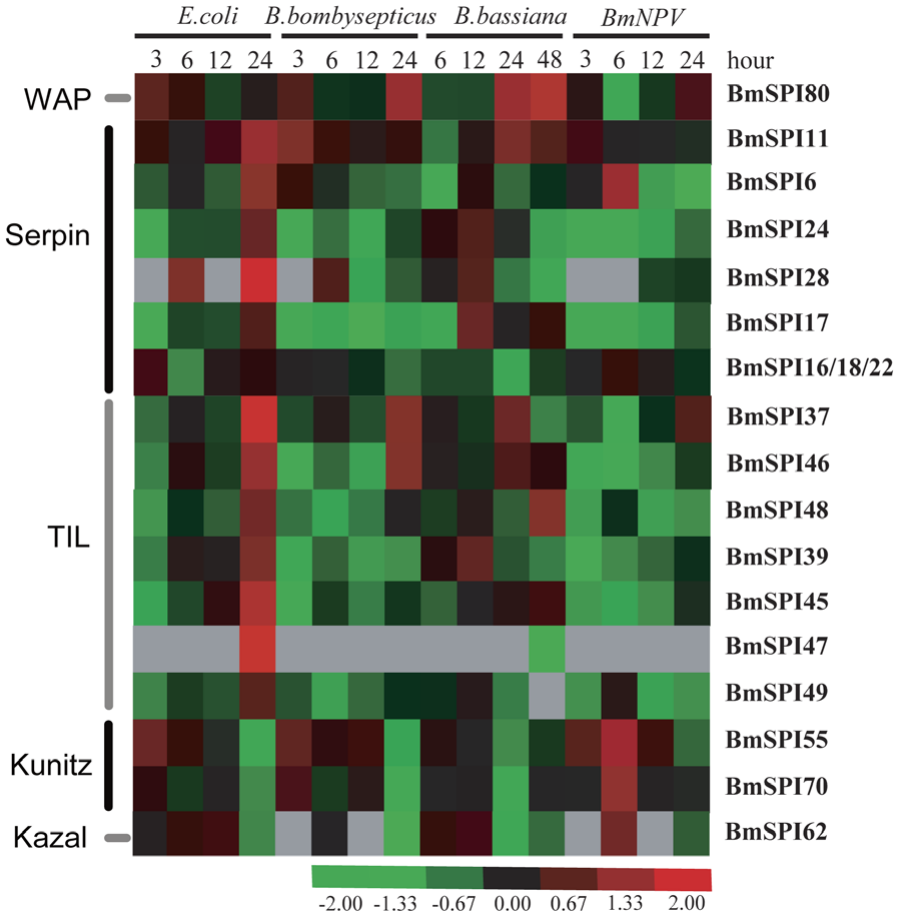
Hierarchical cluster analyses of SPI genes after microorganism infection. Red represents that gene expression is up-regulated; black represents that gene expression is not changed; green represents that gene expression is download-regulated; gray represents data missing. Hierarchical clustering was performed using Cluster version 3.0 and visualized by TreeView.

Many insect SPIs have been considered to be involved in the innate immune response against pathogens. To discover more *B. mori* SPI genes that involved in the immune response, we analyzed SPI expression changes after oral infection with four microorganisms (*Escherichia coli*, *Bacillus bombysepticus*, *Beauveria bassiana* and *B. mori* nuclear polyhedrosis virus). We found that nineteen SPI genes were up-regulated or down-regulated with at least 2 folds, compared to the controls (Table S4 and Figure 6). Eight SPI genes (*BmSPI6, 11, 28, 37, 39, 45, 46 and 47*) were strongly up-regulated 24 hours after infection with *E. coli*. *BmSPI11, 37*, *46* and *80* were up-regulated 3 hours or 24 hours after infection with *B. bombysepticus. BmSPI11*, *48* and *80* were up-regulated 24 or 48 hours after infection with *B. bassiana*. *BmSPI6, 55* and *70* were up-regulated 6 hours after infection with BmNPV. As shown in Table S4 and Figure 6, strongly down-regulations of some SPI genes were also checked after pathogens infection. For example, serpin-type *BmSPI17*, *24* and TIL-type *BmSPI37, 39, 45, 46, 48* and *49* were strongly down-regulated 3∼12 hours after infection with *B. bombysepticus* or BmNPV. Kunitz-type *BmSPI55, 70* and Kazal-type *BmSPI62* were strongly down-regulated 24 hours after infection with *E. coli, B. bombysepticus* or *B. bassiana*. Theses results from microarray data were also well supported by real time RT-PCR examination (Figure S4), suggesting that SPI genes might play important roles in the innate immune system of *B. mori*.

## Discussion

143 serine protease genes have been identified prior to the current study based on the genome of silkworm [67]. 38 SP genes detected in the midgut may be involved in digestion, and SP genes expressed in other tissues/organs may have function in the process of immune, development and metamorphosis. 34 *B. mori* serpins have been identified and form a large family of SPIs [45]. However, there are various SPI families in the silkworm besides the serpin family. This work identified 46 SPI genes belonging to other 9 families: Kunitz_BPTI, Kazal, WAP, TIL, amfpi, Bowman-Birk, Antistasin, Pacifastin and alpha-macroglobulin. 39 SPIs were novel genes and some SPI families were also firstly identified in *B. mori*. This article provides an overview of the silkworm SPIs, and will facilitate future functional investigation.

Except serpin and alpha-macroglobulin, *B. mori* SPIs from other eight families were classified into canonical SPIs, which are cysteine-rich proteins and could form disulfide-bonds to maintain their rigid structure. The rigid structure keeps correct conformation to interact with the active site of SPs [68]. The single domain of cysteine-riched SPI usually has low molecular weight. However, some inhibitors contain multiple inhibitor units ranging from 2 to 15 and are defined as compound inhibitors [46], [69]. The current work identified many compound inhibitors. For example, BmSPI68 and BmSPI49 have 9 Kazal units and 5 TIL units, respectively. Moreover, A few compound inhibitors contain inhibitor units from more than one family and are defined as mixed-type inhibitors. For example, BmSPI56 is composed of 4 types of inhibitor units, Kunitz_BPTI, Antistasin, WAP and TSR domain (Figure 2). Kunitz_BPTI, Antistasin and WAP are canonical SPI units, but TSR is a cysteine protease inhibitor unit. Both compound inhibitor and mixed-type inhibitor consist of multiple active sites and could inhibit a wider range of proteases. It is interesting that some SPIs contain both non-inhibitor domains and inhibitor units, exemplified as BmSPI57 (lacunin), BmSPI55 (spondin) and BmSPI58 (papilin). They are all extracellular matrix proteins with high molecular weight, and may interact with other extracellular matrix proteins in embryogenesis and morphogenesis of various tissues.

Insects rely on innate immunity to protect themselves from pathogens. This research revealed that 19 SPIs from serpin, WAP, TIL, Kunitz and Kazal families were strongly up-regulated or down-regulated after oral infection with four pathogens. However, they showed different sensitivities in response to microorganisms. Serpin-type, TIL-type and WAP-type SPIs could be involved in defense against bacterias and *B. bassiana*, while Kunitz-type and Kazal-type SPIs may play roles in response to BmNPV (Figure 6). Previous reports have found protease inhibitors (PIs) present in the hemolymph of insects from different insect orders [70]. The amount of PI in the hemolymph significantly increases after injection of inactivated bacteria into the hemocoelom of Galleria mellonella-larvae [70]. Canonical SPIs (such as SPIs from WAP, TIL, Kunitz and Kazal families) may directly inhibit proteases from bacteria and fungal to protect their hosts from infection by pathogens [56], [57]. However, SPIs from the serpin family regulate immune response via inhibiting endogenous serine proteases [40], [42], [43], [71]. Insect serine proteases mediate several important defense responses, including hemolymph coagulation, antimicrobial peptide synthesis, cytokine ENF peptide activation and melanization of pathogen surfaces [72], [73], [74], [75], [76], [77]. These processes require cascade reactions of SPs which could rapidly activate immune pathways. However, the activities of SPs must be tightly regulated by serpins because excessive SP activities would cause damage to the host [78]. This study found that six serpin genes were induced in response to pathogens, albeit showing different responses to these gram-negative and gram-positive pathogens. Different pathogens induce different kinds of SP assemblage and need corresponding SPIs to regulate their activities.

This study has systematically studied the classification, domain organization, expression pattern and immune response of SPIs in the silkworm. SPIs not only regulate the immune defense strictly [17], [40], [42], [43], [71], [79], [80], but also control the embryo development and histogenesis through interaction with other proteins [60], [61], [62], [63]. This study also showed that some SPI genes are evolved for the unique physiological functional adaptation. For example, the paralogous genes in the TIL family and Group F of serpin family have generated by tandem repeat evolution (Figure 1) [45]. These SPI genes formed silkworm-specific gene families. More than nine TIL genes and four serpin genes in Group F were expressed in the silk gland (Figure 3). In addition, Kunitz-type and Kazal-type SPIs were also found in the silk gland [57]. These SPIs from different gene families probably co-function to protect silk from being degradated by proteases, or to protect the cocoon under complicate environment conditions.

## Materials and Methods

### Identification of silkworm SPI genes in the silkworm

SPI genes from *Drosophila* and other insects were downloaded from Genbank (http://www.ncbi.nlm.nih.gov/Genbank/). The BLAST tool was from the ftp site of National Center for Biotechnology Information (ftp://ftp.ncbi.nih.gov/blast/). SPI gene sequences from other insects were used as queries to BLAST against the silkworm database (E-value: 10^−6^) [81], [82]. Identified genes were validated by searching against a non-redundant gene dataset and an EST database with threshold of E-value<10^−30^, identities >90%, and match lengths >100 bp.

### Characterization of SPIs

The location information of silkworm SPI genes on the chromosomes was acquired from silkworm genome database. Domain organization and comparison were analyzed by Pfam at http://pfam.sanger.ac.uk/, CDART at http://www.ncbi.nlm.nih.gov/, PROSITE at http://us.expasy.org/prosite, and SMART at http://smart.embl-heidelberg.de/smart. The amino acid sequences were aligned using ClustalX 1.83 [83]. Alignments were manually modified using GeneDoc (http:www.nrbsc.org/gfx/genedoc/index.html). The reactive sites of SPIs were marked out with asterisk.

### Tissue and development expression of silkworm SPI genes via whole-genome microarrays

A genome-wide oligonucleotide microarray with more than 22,000 probes included 55 SPI-specific oligonucleotide probes [84]. Gene expression in multiple silkworm tissues on day 3 of the fifth instar was then investigated. These data were submitted to Gene Expression Omnibus (http://www.ncbi.nlm.nih.gov/geo/) and can be accessed through GEO series accession numbers GSE17571. In brief, The Chinese silkworm strain *Dazao* was reared at 25°C until the third day of the fifth instar. Then testis, ovary, head, integument, fat body, midgut, hemocytes, Malpighian tubules, A/MSG (anterior/median silk gland) and PSG (posterior silk gland) from this developmental point were hand-dissected from on ice. Male and female samples were prepared from the same tissue, each of which was collected from 100 silkworms. The expression data of each gene in each tissue were averaged form four to six repeats. For each gene, if its averaged expression signal was more than 400, it was considered having expression. To determine the developmental expression patterns, individuals were collected for both genders at 20 different time points, including day3, 4, 5, 6, 7 of the fifth instar; start of wandering; 12th, 24th, 36th, 48th, 60th, 72nd, 96th, 120th hour after wandering; day6, day7, day8, day9, day10 after wandering and adult. Three individuals were collected as one sample. TRIzol reagent (Invitrogen) was added and total RNA was extracted according to the manufacturer's instructions.

The microarray hybridization and data normalization analysis were performed by CapitalBio Corp. Procedures were performed as described in detail on the website of CapitalBio (http://www.capitalbio.com). Analyses of tissue expression were performed twice per sample, using a dye reversal procedure in which a female sample was labeled with Cy3 and a male sample was labeled with Cy5. In the second analysis, the male sample was labeled with Cy5 and the female sample was labeled with Cy3. Analyses of development expression were also performed twice per sample, using a dye reversal procedure in which control sample (mixed male and female larvae from the third day of the fifth instar) was labeled with Cy3 and a male or female sample from each time point was labeled with Cy5. In the second analysis, the control sample was labeled with Cy5 and the male or female sample from each time point was labeled with Cy3. Gene expression levels were visualized using GeneCluster 2.0 [85]. The detailed experimental procedures for microarray and data analyses were as previously reported [84]. All the tissue and development expression data of SPI genes is presented in Table S2, S3 and deposited in SilkDB (ftp://silkdb.org/pub/current/otherdata/BmSPI_genes) [82], [86].

### Silkworm oral infection by four microorganisms

For insects used for microorganism induction, newly hatched Dazao larvae were reared with an artificial diet under the condition of temperature of 25°C and humidity of 70%. Dazao larvae were reared to the third day of the fifth instar and were placed in a petri dish without food to ensure hunger before infection [87]. The gram-negative *E. coli*, gram-positive *B. bombysepticus*, *B. bassiana* and BmNPV were used for feeding induction. The microorganism pellets were washed three times with diluted water before optical density (OD) assessment. The artificial diet and microorganism solution (OD_600_≈100) were thoroughly mixed in a ratio of 5∶2 and were then cut into fine grains and given to the silkworms [87]. Each silkworm was fed with about 0.5 g artificial diet and 0.2 ml microorganism solution (OD_600_≈100). After 3 hours, most of the microorganism meal had been eaten by the larvae. Then, the larvae were reared with normal artificial feed at 25°C with approximately 70% humidity. Dazao fed with artificial diet mixed with the same volume of ddH_2_O were used as negative control. Whole body of individual samples induced with *B. bassiana* were collected after 6, 12, 24 and 48 hours, and whole body of individual samples from induction with the other microorganisms were collected after 3, 6, 12 and 24 hours. Three larvae were collected as one sample and three independent samples were got. TRIzol reagent (Invitrogen) was added and total RNA was extracted according to the manufacturer's instructions.

The microarray hybridization and data normalization analysis were also performed by CapitalBio Corp. Analyses were performed twice per sample, using a dye reversal procedure in which cDNA from the control was labeled with Cy3 and cDNA from microorganism induced was labeled with Cy5. In the second analysis, control cDNA was labeled with Cy5 and cDNA from microorganism induced was labeled with Cy3. At least two independent replicates were performed. Genes were considered to be up-regulated or down-regulated if the change in gene expression was greater than two fold on at least one occasion over the four time points in any of the four microorganism induction experiments [88], [89], [90], [91], [92]. Hierarchical clustering of gene expression patterns was performed using the Cluster (version 3.0) and TreeView programs [93]. Ratios of microorganism induced SPI genes are presented in Table S4 and also deposited in SilkDB (ftp://silkdb.org/pub/current/otherdata/BmSPI_genes) [82], [86].

### Tissue expression analysis of silkworm SPI genes by semi-quantitative RT-PCR

Total RNA was isolated from nine tissues (testis, ovary, head, fat body, integument, midgut, hemocytes, Malpighian tubules and silk gland) from day 3 fifth instar larvae using TRIzol reagent (Invitrogen, USA). Contaminating genomic DNA was digested using RNase-free DNase I (Promega) for 30 min at 37°C. RNA samples were diluted with RNase-free water and stored at −80°C. The concentration of total RNA was estimated by measuring the absorbance at 260 nm. Total RNA (10 µg) was reverse-transcribed into cDNA using M-MLV reverse transcriptase (Invitrogen, USA) at 42°C. All cDNA samples were normalized using *B. mori* actin A3 as an internal control (forward primer: 5′-AAC ACC CCG TCC TGC TCA CTG-3′; reverse primer: 5′-GGG CGA GAC GTG TGA TTT CCT-3′). All SPI primers for semi-quantitative RT-PCR detection are listed in Table S5. PCR amplification was performed in a total reaction volume of 25 µL containing normalized cDNA, 10 pmol of each primer, 2 mM MgCl_2_, 0.25 mM dNTP, 1× buffer and 2.5 U of Taq DNA polymerase. Semi-quantitative RT-PCR reactions were performed in a volume of 25 µL using the following program: initial incubation at 94°C for 4 min, followed by 30 cycles of 40 s at 94°C, 40 s of annealing (56°C), 45 s of extension (72°C), and a final extension at 72°C for 10 min. Aliquots of 4 µL of the PCR products were separated on 2.0% agarose gels and stained with EB.

### Induced expression analysis of silkworm SPI genes by real-time quantitative RT-PCR

The gram-negative *E. coli*, gram-positive *B. bombysepticus*, and *B. bassiana* were used for feeding induction. Dazao was fed and oral infected using above methods. Fatbody of individual samples induced with microorganisms were collected after 6 and 24 hours. Quantitative RT-PCR was performed using an ABI PRISM 7000 sequence detection system (Applied Biosystems). The 15 µl mixture included 1.5 µl of cDNA, 0.5 mM of each primer and 1× SYBR Premix Ex Taq (TaKaRa) in each well of a 96-well plate. PCR was carried out with initial denaturation at 94°C for 10 s, followed by 40 cycles at 95°C for 5 s and 60°C for 31 s. The primer sequences for all genes are listed in Table S6. Relative gene expression data were normalized against Ct values for the housekeeping sw22934 gene in silkworm and the fold change (2^−ΔΔCt^) was determined by comparison with average expression levels for control samples, with the index defined as 1.0. Four larvae were used for each treatment. Each expression assay was repeated three times. Student's t-test was used to evaluate statistical significance (P<0.01).

## Supporting Information

Figure S1 **Nucleotide sequences of** ***B. mori*** **serine protease inhibitors.** Overlapping EST sequences were assembled using the SeqMan 5.01 program (DNASTAR, Madison, WI, USA).
(DOC)

Figure S2 **The chromosomal distribution of serine protease inhibitor genes in** ***B.mori*** **.**
(TIF)

Figure S3 **Sequence alignments of serine protease inhibitor domains.** Alignments were done using ClustalX 1.83 with default parameters and shading was done using GENEDOC. Identical residues are shaded black, while similar residues are gray. Asterisk represents the predicted P1 position. (A)TIL, (B) Kunitz_BPTI, (C) Kazal, (D) Antistasin, (E) Pacifastin, (F) WAP, (G) amfpi.
(TIF)

Figure S4 **The induced expression analyses of silkworm SPI genes by quantitative real-time RT-PCR.** We chose the time points of infecting 6 h and 24 h to do the expression analysis. The expression of SPI in the control sample was set to 1. The abbreviations are used, the *E. coli* infected sample (A), the *B. bombyseptieus* infected sample (B) and the *B. bassiana* infected sample (C). Each expressive assay was replicated three times. The Student's t-test was used to evaluate statistical significance (* P<0.05, ** P<0. 01).
(PDF)

Table S1 **The serine protease inhibitors in the silkworm.**
(PDF)

Table S2 **Multiple tissues expression data of SPI genes.**
(PDF)

Table S3 **Developmental expression data of SPI genes.**
(PDF)

Table S4 **Ratios and annotations of microorganism induced SPI genes.**
(PDF)

Table S5 **Primer sequences, sizes of PCR production and melting temperature for semi-quantitative RT-PCR.**
(PDF)

Table S6 **Primer sequences and sizes of PCR production for qRT-PCR.**
(PDF)
