# Supplementary material for: Genome-Wide Identification and Immune Response Analysis of Serine Protease Inhibitor Genes in the Silkworm, Bombyx mori
Source: PLoS One. 2012 Feb 13;7(2):e31168. doi: 10.1371/journal.pone.0031168 (PMC3278429; doi:10.1371/journal.pone.0031168)
Supplement: Figure S1 — Nucleotide sequences of B. mori serine protease inhibitors. Overlapping EST sequences were assembled using the SeqMan 5.01 program (DNASTAR, Madison, WI, USA). (DOC) [file pone.0031168.s001.doc]

>BmSPI80

ATAGGATATTAAGTTAAAATAGTGTGTGTGTGTGGACTACGAATTTGTCGTTATAATCTCAGCTGTGTTGCGAATGACCCGTGAGTTCAAGAGTCCAGAAATGGTGCGTTGTTTACTTCTAATTATCGTGTCCTGCCTCACATCGTACTCGCTGTGCGGTTCGTGTCCTCCGACGTTGTCCGTGGACATTTGCGAGCCAATGTGTGGTCCCGGTCAGGAGTGTAACGGGACTCAGCTCTGCTGTCCCACGCACTGCGGTGGAGCCATGTGTGTTGATGCGATGACTCAGAGGCACTTTGTTCATTTAGTAAAGAAAGGTAATTGCCCCGAGTTTCCGCGCGGCCCGTGGATCTGCTCCCACACTTGCACCGGCGACTCGGACTGCCCTCGTGCCCTCAAGTGCTGTCACAACCGCTGCGGGGTCCTCACCTGTCAGAAGCCGGAAATAGATCCCGAACCATTTGTTGAACTTCCATAGATCTAATTTTGAAAATACATAGGTATATATCTACAAAAAGAGTGTTGCCAGAATTAGGTTTTATGGAGAACCGTAATTATAAGTATACCACAAAAAAAAAAAAAAAAAAAAAAAAAAAAAAAAAAAAAAAAAAAAA

>BmSPI79

ACGAGCGCATTTCAGACGACATAGACGACTATCGCCCGTCGTAACTCCAACGGGGTCAATGACATCGATATCGATTATGTTACGGTTTTAATTGATTAAAATATAATCGATCATCACACGAGATTTTAAGGTGAAGCCGGATTAGTGTTTTGATGCAGGAAGTAGTTACATTTATGGACCATTTATTGTTTTATCGTGAACGCGAAATGTGCTAGAAACAAAATCTTTTTGTGTTTGGTGAAAATGAGCTCAAAAATGCTGATGAGACTGTTGACGGTGATGGTGGTGGCGTCTCTCGTTCCTGCGATCCAAGCGAGGGCGCGGAGATACTCGAGAATGCAACCTAATGCTTTATCAAAGACAAGATGCGATCTGATGTGCTTTGACAGGGATAAGGAAAcTAAaGGAACgTGTCGTTCCCAATGTCGGAATCAAGAGCACAAGCCGGGAAAATGCCCCGTTTCGGACACGCCGAAATGGGAAGCCGCATGCGTGCAGGCCTGCAACTCCGACTCCCAATGCGACGGCACACAGAGGTGCTGTCACCACGGATGCGGTTCTACTTGCAGTGAACCTTTAGACTTGTTGACTTTACCAGGTCTTCCAGCCGTTCCTACAATGGAAGAACCAAAGGAGAAGCGACGAGCTGTCGTTTTGCGCTGGTCAGACGGCGTAGGAGATACAGCGAGGGCTGTTCCAGGACGAGTCCTTTACCTGCTTGAGGAACAACATCATTTGGGCCCTAAATATGAGCAGTCTAGGCTTGGCGATTGGAATCTCATGTTACGTACTAACAGGACAAAGGTTTCGCYGAGGAATCTGTTGAAACCAGGTCGCTGGTATCGGTTCCGTGTTGCTGCTATTAGYGCAKCTGGTACACGAGGGTTCTCCGATCCCAGTCCYCCATTTACACCACGTCGTGGACCCCGTCCTCCTCCTATGCCGAAGAAATTGAGAGTCCGACCAATGAGAATGGATAACGGAACGATGACAGTAAGACTTGAATGGAAGGAGCCTCGTTCTGATCTGCCAGTGATGCGCTACAAGGTATTCTGGAGTAGACGAGTAAGAGGATTGGGCGGAGAGCTAGATTCCGTTCTTGTCAATCATCAGACAGTTTCAAAGGWTYRAAACCACATCGAAATAAAAGATTTACAACCGAATTCTATGTACTTTTTGCAAGTTCAAACGATCAGTCAATTTGGTTTGGGTAAATTGAGAAGCGATAAGGCATCTGTATTTTACAACACGACTGGTTCTGTAGGAAATGATTCTGTACCAGAATCTTTAGTCAGACGTGACAGATATATAAAGGGTTTGAAGTTGAATAAAATTGTTTGGAACAATCAAAGACTGAAAGCGAGAATTTCATGGGAATCAGTGCCTAGTGGAAACAGTGGCGGAAGGTCACAAGAGAGATATTACGTCCATTGGAAAACTATAAAATGCAATAAAACAGATAAACCCATGAAAGACTTAGCGGCGACCACAGCGCAATCTACGTTTGAACTCTATGAATTGGATTACCACTGCAGCTATAAAGTAAATGTGAACAGATCCTGGAAGAATAAAATACCAGAATCAGAGCTCGTAATAACAATTCCTAGATGTCAGTATTTTAAAAGAAAAGTTAATGTTACAGCCATAACGTGTGACTCGTAATTTTCAAATTTCGTTTTTTTATTATAATAGTAACATTTATAATATGTATTAATAGTACTAATTCTACTTAATAACTTTGACATTAAATGTATAGATATCATAGACAAGTTATTTTGAAACTTAGATCTATTTAAATTGAATAAAATTGCAACAAAATGAGATTAAGACTGTTTTCGTAACTAATCAAGTAAATAAATGTATACGGAATCATTATTATAAAAAGACTTAATTCTAG

>BmSPI70

TTTCACATTAGGCGACGTGCTGAGCGCTCGCTGACGGCTGTTGACTCGTTAATTCGCATCATTTATTATTGGTAACGGAACGTGCCAGAGCTTGAAAACGAATTGAATAAATCGTTCCATTGAAAATAAGCAAAACAGTAACAATGTCGCTAAAGCGGCAGCTCTTAATTTTATTCGGGAGCATTGGTCTCCTGTCTGTGGTACATGCTGCCACAATTACGACCCCAGAGACCATGGTGGTGTCCAGATCGGATGATGCGGAAACTGAAGCAACGCTAGATGTAGATACAGTAACGGAGGGACCTGTTCAACCAATAAAACTGACGAAGATGGATGTGAAGTCCGTGATATCTATGCGATACGCTCACACGGCTATTGTAGCGCATGTGCGAAATGCTGCAAACAAATCTCAAGAAGCCAATTTCAGGGTGTTACTCCCCGACACAGCTTTCATCAGCGGATTTGTTATGACTCTGGATGGAAAATCATACAAAGCGTACGTCAAAGAAAAGAAAGAGGCTGCTCAAATATATCAGACAGCGGTATCACAAGGAATTGGTGCCGCACACATCGCTGCCAGAGCACGTGACTCGAACCATTTCACTGTATCCGTGAATGTGGAGGCTTTCACCAAAGCCACATTCAATTTGACATACGAAGAGTTGTTGGAGTATCGGAACGGTGTTTACAATCATGCCGTCAATCTGCAACCAGGGCAATTGGTTTCCGATTTCACTGTCACCATTGACATTCGGGAGTCTAACAAAATCACCGAACTGAGGGTGCCGGAGATCAGAACCGGAAATGAAATCGACGCTACAAAAGATGACGAACAAATTTCTAATGCAGAAATTACCCGGTGCGATAATGCAGCTGTAATTTTGTTTAAGCCCGATTTAGATGAGCAAAAAAGATTGATGTCAGTCTATGCAGAAAAATCCAAAGACTCCCTTCGGGGTAATGTTGAGGGTGTTCTTGGACAATTCGTGGTTCAGTACGATGTTGAACGACCAAAAGACGGCGAAGTTCTTGTAAATGGTGGTTACTTCGTCCATTTCTTCGCACCGACGGACCTGGCACCGCTTCGTAAATATGTCGTTTTCGTCTTGGACACGTCAGGATCTATGTACGGACGGAAGATTGAACAGTTAAAACAGGCGATGCAGACAATCCTAAGCGAACTAAACCCCGGAGATTACTTCAGCATTCTATCCTTCGATTCTGATGTATTGGTGACTGACATAGCAGATGCCGATAAGGAACCACCGAAACGAAAGTATTCGTACTACGATCGTCACGATGTGAAACCGACGCTAAAGCCGGCATCGAAAGCGACTCCGGAGAATATAGCACGAGCTAAGATCATCATCGACAGACTTGAAGCAAACGGAGGTACCAACATTGATGCTGCGCTCGGTACTGCCATAGATCTAATCAGGAACAGATCAGAGCTTTTTGCCAATTCAACATCTTCCAACAAAGATGAAATCCTGTCTTTGGAGCCCATCATCATATTCCTGACGGATGGCGATCCGACTGTTGGTGAAATGAACCCGAAAACAATAATTAAAAATGTCGCTGAAAAGAATTACGGAAGTGATGAAGCCACGATATTTTCACTTGCTTTCGGTGAGGATGCGGATCCCAAGCTCCTGCGCAAGTTGTCGCTCCGCAACAACGGTTTTGCGCGACACATCTACGAGGCCTCAGACGCGGCGCTGCAGCTGCGCGACTTCTACAGACAGGTCTCCTCGCCTCTGCTGTCACATGTCAAATTTGTCTACGCACCGGATCAGGTGATCGAAAGCAGTGTGACCAAGACCAAGTTCCGTACGTACTACGCGGGGTCCGAGGTGGTGGTCGCCGGGCAGGTCGATACCGCGACGCGGGACCTCGAGTCCACCGTCGAGGCCTTCTGCGGCGATGAGAACGACCACTTCCTGAGGAAAAAAATCACAAAGAAAATCAAAGTTCCGGTACCCGTCGAGAAAAGTGATTTACCCCTTGAACGTCTATGGGCCTATCTCACTATCAAGCAATTGCTCGACGCAAGCGATGCCTCCGACGCTACTGAAGAAGAGAAGAAGAAAGAGACAAGTCCTGAGCAGCAGGCCCTTAAAATAGCTTTGAAGTACGAGTTTGTGACGCCGCTAACTTCTCTGGTGGTAGTGAAGCCTAACGAGACGGACGCAGTGAACGCTGAGCCGGTCGGCGATCGGGAAAGCGGTTCGCATCGCCAACCCATCAGTAGCCCAGCCATGTATCGGCCGTCACTAAATGCAATGTCACCTCTTGCCTTTTCGGCCTTCCCAAATAGAGGTTATACTGCATTTAATAAAAGGCCTGTTCAAGCATTATTTTCACAAGATTCAATGAGAGTGGAGTCGGAGGCCGTTGACGATTACGATTATGAGCAAAGATTTGGTGTGGATCCTCCATCGACTCCTGTTGCCTATGCACCACCAGCATGGTCTACAACGCCGGTGATCGAACAATCAACCACCAGCAACGGCAGCAACTACCATTTACAAGAGTATCCCTGGGCAGCGCCCTACGTCGATGTCTCCAGCGATAGCTTAGTTTTTGTACCCGCCAATAACACTATCACATTAAAACTAGTTAGCTCATCTGAGCCTCCTAAATCAGAAAGTGAATGCTCTAAGCCAGTGAGCGGCGACGCGGGCCGGTGCGTGTATCTCACACGCTGTCAAGCCGCTCGGAATATCACCATCGACACCTACAAATCTCACTACTGTGACGTCGCTGGGTTCGCTGGAGTTTGCTGTCCCCAAGACGGCATTGCAGTGTAGTAAATTTGTATTATTATATCCTATTCTGTTTGTTACCGCGTCTGTTTTCACATAAATTTTCAATGAATCACCGTTCACGGGCTTGTTAGACCTCAAATAATGTAACTTTTTAATTGTACTCTTTTCTTAATTCATCACAATAACGTAGTTTCGAAGCCCAGAATTTATATTTCATTATTATTATTTGTTAATACGTTTACATTACATAATTAAGTATTGTATGAAATGATAAAAATAAAAAATGCTAGAAGGTAATTTCAAAATTCATTTAAGAAATAAAGTAAATATCAAAACAAAAAAAAAAAAAAAAAAAAAAAAAAAAAAAAAAAAAAAAAAAA

>BmSPI68

GAGTTGCATTGCGTCCGCCATCGACGCCGAACGTGTGGCCCGCCCCTTACATCCACCACGCCACGATCGTGCGCCGCGCCGCCTGCTTGCATTCACTCAATCTAGATTAACTTCGAGTTCTAGTGCTATTGTGGTGATCTTTGAGGCGGCTTGCTGGTTGAGCTCTTGTATCGCGCAACATGTGCCGCAGGAAGTGAGCGAGGACAACAGTGCGAGACTAGTTCTGTTGCGATACTCACAGAAGACTCACTTGCTAGACCGTGACCAGCGTCGCCGCACCATGGAGACCGGGACCTACCTCCTCGCCGCTGCTGCCCTCCTTGTCCTCGCGCCGACGAGCGAGGCAGCTCGCGACGCGTCGTGTCCTCGCATCTGCGGGCCGGCGCTGCAGGGGGAGCCCGTCTGCGCCACCGACGGGTACATCTACCCCTCGCTCTGCGAGATGAGGAAGAAGACTTGCGGGAAAGGAGTGCGGCTAGCCCCGGACCAGGGCTCATGTTCCCGCGCGCAGGGCTCCAAGTGTGACCACCGCTGCACGTCGGAGCGGGACCCGGTCTGCGGGACCAACGGACGGACCTACCTCAACCGCTGCATGCTGCAGGTCGAAATCTGCAGGCTCGGCATAGGCCTGTCTCACCTGGGCGCCTGCAACAACATCAGCGCGCACCGCGAGAACTGCCCCGTCGACTGCTCGCAGGCCCCGCTCGACGGCCCCATCTGCGGCTCCGACGGCAACGTGTACAAGAGCACGTGCCAGATGAAGCTGCTCACTTGCGGACAAGGAGTGGTCCGCACCAGCAAGAAGCACTGTCAGACGACCCGCCACTGCCGCGAGTCGTGCTGGCGGGCGGCGCGCCCCACCTGCGGCTCGGACGGGAAGCTCTACGCCAACGCGTGTCGCATGAAGGCTACTAATTGCGGCAAGCACGTGTTCGAAGTACCGATGGCGTTCTGCGTGTCACAAGAGCGCACCTCCGGGGGAGAGTCCTGCTCCACCGACTGCTCCGGAGAGAAAGAGAAACCCGTCTGTGGCTCTGATGAAAACATTTACAGGAATGAATGCGAGATGAAGATGCTAAACTGTGGGATAAACAACAGGAAGATGGTGAAGAGGGTGGACATGGAAAAGTGCAAGTCCAAAATGAACAAGTGTCTGAAGGTGAAATGTCCGAGCGACGCGGACCCCGTGTGCGGTACCGACGCTATCGTCTACGCCAACTCCTGCCACTTGAAGGTCGCCACTTGCTTACGAGGAGTTCAGCTGGCTCACTTCGGAAACTGCACGCTTCTGCCGCGCCTGGAGACCGACTGCCCCGACAACTGCGACAACGTGCTGGAGCAGCCCGTCTGCGGATCCGACGGAAACGTCTACAGGTCTGAGTGCGAGCTGCGGCGGTTGACGTGCGGGCAGCACGTGGTGGCGGTGGCGGCGTCGCACTGCCGCACCACGGCGCTCTGCCACGAGCACTGCCCCGACACGCCCGCCTTCATCTGCGGCTCCGACAACCGCTTCTACAAGAACGAGTGCCTCATGAAGAAGGAGAACTGCGGCAAGCACGTGTTCGTGGTGCCGCTGAAGCGCTGCCTGGCGCGGTTCCAGTACGCGGGCTGCGCGCGCGTGTGCCCGCCGGAGTACGACCCGGTCTGCGGGACCGACGACAAAACCTACTCCAACAAGTGCTTCCTCGAAATGGAGAATTGCCGCTCGAGGAGTCTCGTCCAAATGAAGTACCTGGGGACGTGCTCTGAGCCGATCGCGGAGGAGCCCAAGAACTATCTGTATAGGTAGAGCGTGGAGTCGAGACAGTGATGCATTCTTGCTTCAATATTGTAATCGCGTCGTGGGATATTCAGTGCCAACTTTACTTCTTGCTACACTTCTATTTGAAATAACTCGTCTTCCACTTTATAAACTTAGATTTATTATGTAATTATAATTACAAATTTATTTATTAAACTCAGAGACGCCGTAGCCTTGCAAGGCTTTGACGGTATATGTATCTGATAAATAAACTTTAGATTAGTATCACTAATTGTCTGTACGCAAATATGTATGTAATGTTCAACTAGCAAACGTGGTTAAATTAGCAAGTACAAACTATGTATCTGAACACAAAGCGTTGTCGACAACATGGCGGCCGCGTCCCGCAAGCAAGTACCTGCGGAGCGGCGAGCGCGTCATACTCGACGGTGGGTGACGCGGCGGTGGGGACGCGACAGCGGGTGACGCGCTGGCGGACGCGGGCCACTACATGGCAAACTATTTATTGTGTAGATTAAGTTAACTCTTTGTAAATAATTAGCGAATTGTTTCTAAATAATATTTATTTATTTACGTTTTAAGTTTTTTAATGCATTTACGGAAAAGACTCAGGAATTTATTAATTTTAAATTATTACAGTATACTGTCAATCAGACCCCGGTAGTGAAGCGCGGGGCTGGTCGTGCGGTGTCGATTGTGTAGAAGTGAACAATAAAATTGAGTAATAAACATAAAAAAAAAAAAAAAAAAAAAAAAAAAAAAAA

>BmSPI65

TCGGCTTTCTCACAGCGCGAGTCAAAAAGACTTGGACTTAAAAATATATATTAATTCACAATGTACTTCAAAATAGGAATGTTACTAGCAGCCACGCAGGTGATTGCAACACTTGCATATCCACCAAGCTGTGCGTGCTATAGAAACCAGCGACCAGTGTGCGGAACTGATGGGAAAACATACAACAACGAGTGCCTGCTCGACTGTGCCACCAGAGATGATCCCGGTCTAAGGGTCAGATATCAAGGACCCTGCTCGGAAGGAAACGTCGGCTTCCCCGCGTGCCACTGCGACTACGACCTCAACCAGGTGTGCGGTAGCGACAACCACACGTACGACAACGCTTGCCTGTTGAACTGCGCCGCAGCCACGAACCCAGGCCTTAGCATTTTGTACTCGGGCCTTTGCGCAGACGAAGTCAAAATCGTGGACGGCCCCAGCAAATACCCTTCGTGCACGTGCACGCGCGAGATGAAGCCGGTCTGCGGCAGTGACGGTATCACGTACAACAACGACTGCCTCTTAAACTGTGCCACAATCAATGACTCCAGACTCGGCATCGAATACTACGGGCCTTGCGCTGATAAGGTTATAGTCGTCGACCCTGGGACGCAAGGGGACTACCACGGAATACGACCCCTGTAATCGAACTGCATACGCCTCTCGTACAAGTCTTATAGCGTAGTTTTAGCGCAATGTTTAGAACGACAGATGACCGCCTTCATAGAGAAAAAAAGACCTTTTTTTTATTTAACAAAATCCCACAAGACTTTGG

>BmSPI62

TAAATGAATTGACGTCGCCGCTAATGTTTACATTAATACGGTTTTGTTTGTCGAGCAACGATAAATTTGAGGCACGCTACGGGTATGCTTGGAATGAGAATTGACGATTTACCGACTCATTGAAGACAAAGCTGTGATAAAGGTGGACGAAGGCTGTGCAGATTGGTATGGGCGTGCGGTGAGATTCGGAGCACTCCGCCAGCATGTTGGAGGGAGAGAAACAGCAGAATGCAGGCGGCACCAATGCCGCCCCACAGCACAGAAAAGGACACAGGAGGCAAGAGTCAATGTACGCTATGACAGGACTATACGCCGAATCAGTTGGCATAGATGGCGAAAAGGTTGCTAGGCCGATTACGCCACCTCTACCCTCGCATCCACCACGGGAATCTATCAAATGTCACAGCCGGAACCCCTCCGCTGGTATATGCGACAGGGATCGCGAAAAAGAAAGAGAAAAACCCCGACAAATATTTCCAGAAATACTGGACATTCCTCATGACGCAAGAGATTGCGGTATCTTGTCTTGGAGGCCGCTACTCATTCAGAGATTTTCTAGTATAAAAGTGTTTGTGTTCTTTCTTTCTTTTCTTGTGACGCTTCAACAGGCGTTGAGTTCCGGCTACATTAACTCTGTGATCACAACTATTGAAAAAAGATTCGAAATCCCTTCGAGTCTTTCTGGATTAATCGCGAGTAGCTACGAGATAGGAAACGTCATAACAGTGATTTTCGTTTCATATCTTGGTAGTAGACGACACATTCCAGTTTGGATAGCAGTTGGTGCTGTAATAATGGGTATCGGGTCGTTGGTGTTTGTAGTTCCGCACTTTATTGCGGAAGTCAATAGCGAAACTTTGGCAAACAATCAATCGGAAGAAAACATCTGTCGGCTAACACATGCCCTCGACCAAGACATGGTCGGACGTTTATCAGTACAAGGCCTACCGCCAAACAATTTGAGACCAGATAACTGTATTAAGAGCACTCCGAGCACATTCTTGCCAGTAATGGTGTTCATAGTTGCACAACTATTGCTCGGTTGCGGCGGATCGCCATTGCTGACGCTTGGCACAACTTACGTCGACGATCACGTACGCCCTGAATCTTCTAGCATGTATATCGGATGCATGTACAGCATGGCTGCTTTCGGCCCTGTCCTCGGCTTTTTGCTTGGTGCCTACTTATTATCTTTTCATATGGATTCGTTTTCCGGCGCTATTATATCGATTGGTCCCGGTGACCATCGTTGGGTCGGAATGTGGTGGGGCGGTTTCCTACTCTGTGGCTTACTTCTGATTCTCGTCGCGGTTCCGTTCTTCTCTTTCCCAAAAGTCTTGGTTCGAGAAAAAGAGAAAATCAGACTTGTAGAGAAAGCGGCCGCCGCTAGCGGTGCGTCGACTTCTAAACCACCGCCCAAACCACAAACCGATATTAAGGACTCCGGCTATGGGAAAGACATAAAAGATATACCGGTGTCGATGTGGAGGCTGCTAAAGAACCCCGTATACGTTGTCACGTGTCTCGGTGCTTGCATGGAACTCATGATAGTGTCTGGTTTCGTCGTCTTCTTGCCCAAATATTTAGAAACGCAGTTCAGCCTCGGCAAAAGTCAAGCCAGTGTTTTTACCGGCTCCGTCGCCATACCGGGTGCGTGCATAGGAATCTTTATGGGCGGATGTTTACTGAAACGTTTGGAGTTGCGACCCAAGGGCGCCGTACAGTTTGTGCTCATATCAAACATCATTTGTCTCTCTTGTTACGCGCTACTGTTCTTCTTGGGCTGCGACAACATTAAGATGGCGGGAACTACAATTCCTTACACCAACAACAGTAATCTGGAACCTTTCAAAGTGAATCTGACGGCCGCGTGCAATTTAAACTGCCTGTGCACGGAGACGGACATGGAACCGGTCTGCGGGAACAACGGCCTCACGTACTTCTCGCCCTGTCACGCCGGATGCGCCAAGTTCTCCTCGCATCGGTCCAATTTCACCAACTGCGCATGCGTGCACGAGAACAGTATGGGCGGCGGCGGCGTGGTGGCGGCGGCGCTGCGCTCGGACTCGCGCGCGCAATACAGTGACGTCACCATCGTGCCCGTGGCCACGGCCGGCCCCTGCAACCCCCCCTGCACCACCATCTTCCCCTTCCTCGTGCTGCTCTTCTTCATGACGTTCGTTGTGGCCGTTACGCAGATGCCGCTCCTCATGATCGTACTCAGATCTGTGAGTGAAGAAGAACGTTCGTTTGCTCTCGGTATGCAGTTTGTGATATTCCGTTTGTTTGGATACATACCGGCCCCAATACTTTTCGGTAATCTCATCGACTCCACGTGTATTCTGTGGAAACAATCATGCAGCGGCGAGAAAGGCGGACGTTGCCTGTTGTACGATATCGAGCAATTCCGATACAGGTATGTAGGCTTGTGTGGAGGAATAAAAATCGTAGCTTTAGGCATCTTCCTAGCAGACTGGTGGTTGGTTAGAAGAAGGAAAAATCTCGAGACCGCCGCACCGCTCGATCCTCACAAAGACATCGCCGGTTCAATCATCAGCCTCGACAAACTATTCGAGGAGTTGCCGTCGGCGGAGAACGCGAGCGGGTTCCGGTCAGGCGTGACGTCAGGACTGAGCTCGGCGAGCAGCACGCCGCTGGAGCCGGCCGCGGCCGAGAGCCTGCAGCGCGTCGACTCGCAGTACAAGAACTCGCGCGTCCTGGTCGCGTCGCGCCATCTCCGCAACGACTCCAAGACCATCCAGCTGGAGCCGCGCACCCGCCAGCGGTCGCTCGACGAGTCCGAGCGAGCGTTCCCGCGCTCAGCCTCGCGGGACTTCCCCGGACACTCGCGAAACGGATCGCGCGACTTCAAGGTGCACTCGCGATCGGACTCACGCGATCTCAGCCTCGACCAGCTGAGGCAGCTGGCGCTCCGCAGCGTCGAAAGCCTCGATCTGAACGTGCTGCCGCTCGCCAAGTGTGCCGACGAGGAGAGCAAGCGGCTGATAGAGAGCGGGGGCGTATTGCGCCACCGGCGAACGAGTTCGAGGGACATCAAGCCGCCAGAATCGAAACACAAACGGACTTCGTCACATCACATCACAATGGAGCCAAATGAACTCAGCCTTCAGATACAAAAAGGACGTAGCGTCGATCACCTGGCATCCGCGCCTCTAGAACCCCGCGTGTGAGTCAACCGCGAGTGACGATTGCGTCGCGGAAGGAATCTCAAACGGCGTCGAAGCTCCATGAGGAAGCTTTGCTGGTCGCTTTATGGCTTGACCTCGTAATGCAAAATAGTAAAATTCAGCTTCTGTAGACTAATTTTATTTTGACTACTTAGATAAATAAATGAGTGATTTCAAATCATATCCTAATGGGTCGTGTTATATTGTTTTCTTATATTTCAATGAAAAACGTTTTGAAGTTAAATATATATTTAGAAGAAAAAAACTGCATTGATGTGATCAAACACACTAATTGTGTTTATTATTAGCCATGGCAGCAATTTTTAGCATCAAATTACTGTACTTAATACTTTTTAAAACAGATCTTCTCAATTAAATCCGTGGAAGTTATTAAAAAACTTTTAAAACATTTAGTGATTTTAGAAAATTAAAGTTGAATCATTTTTATTTTAATATACCATTGTGACTACTTTGTATTTCAGTGCTCTGTAGTTAAACCAATTGAAATATGTCTAGAATAATAATTCCTATTTTAATAAATGTTTGCATGATCATATTATTGAAAATAAATCACAACTATGTAGATGTTACTATAGAAAGAAAAAATATCACTTAAAGTAGACTGATGTGAATATCACAGTAAAATTTTTAATTGTAAAGGTTGTCTGTAATATGCCACAAATTATTAGTAGACCGCAATATTGTGATATGAAATAATTTTTAATTCTAACAAAAAATTTTAGAAATAGGTATGTCTTGAGAGCAGTGGGATCACTGAACCAATGCCATTTATGCCTTATTTTGCTATAAATTTCAAAGTTTAAATGGAATGCATATATACTTGAACATTTTTTAATTGTATTGTATTTTGACTTACTCTGACATTCCTATTAAGGATCAAGGATATATGTTGCCATAATAATATGTCACATTCAAATTTTGTAACAAATGCTATCTCACAGCAGAATAGGCATTACTCCTACACAACTGAGACTTCAACCGAATGTATCAAACGGTCCATTGCTATTCAGTGTATTGTCAAAGGGCTCCAGTAAACTATTTACATCAGATGCTCCATTAACTTGTCCGCCCATTCAAAAAAAAAAAATGTGTATAATGGTTTATGGATTTTGAAACGACAAAATTTAAGTGACCGATACATTTACAAAATATGTAAAAGTTCTTAGCTTTTCATATTTTAGATTTCCAGAATTTGTTGTCACTATGTCATGTGTTCATATTTACCATTTTTTTATTATTACCTACCATCCACTACAATAAGTTGTCTTGAACAGCAGCATAAACAATATTTTGCATATCATGTCTGTTATACTGGTTACATATAGAACATAAAAATATGAAATACAAGCATCTTTTTTAGTTATTTCTTAAAATTGATGTATTAAAAAAGGTGTCAATTGTCAGGCTACTCCTTTGGTACTAGAAATCAATTAAAATTATATAAATTTATTTTAATAGCAATGTAAACACTAATATGAAGCAATATAGTATTAAGAAATATATATCAGAGATATATTTAATTAAAAAAGACAAGAGAGATGTTGTCTGTGTAAATTTTGTCACATATATGTTAAGAGAGGTTATAGTTTCATATGGAAAAAAGAGTGTTTTACCTAAAATGCCTTACTACAATAGGTCTTAAGACTTGATAGTGATACTGACTTAGGTGTATGTAAATGAATGTTGTTATGTCAATCACACAGATAAGTACGATGTTAACAACTTCTAAAATAATTTGTTGCATTTAGTGAATAAATTAAAATTGCATTCTTAAAAAAAAAAAAAAAAAAAAAAAAAAAAAAAAAAAAAAAAAAAAAA

>BmSPI55

AGTACGTCTTATGCCCAAATAAAATGGGCAATCTGAGAATTTTAGTGTGGCTGGGCCTAGTGTCCGCGGCACTGGCCTGCGAGCTAAATCCAGGTCCTGGAGTAGGTTCAAAGTCACCTGGAGACAATCACTACAGGCTGATTGTTAACGGAGAAGTTGAGCGATATGCGCCGGATCAGAGATATGTCGTGACTCTGGTCGGTTCCCGCACTCATGACGTAGTACAACAGTTTGCGGGCTTCAAAATAATCCTTGATCCTTTGAACCCGGACACAAGGAGGGCGCCCAGCAAACAGGGACAGTTCCAGCTCTTCGCCGATACTTTAACTAAGTTCGACGAAGAATGTACGAACTCCGTTGTCGAGGCTGACGATCTGCCCAAAACTGAAGTCCAGGTCATGTGGAAAGCGCCACCGGCTGGTTCAGGATGCGTGCTTTTGAAGGCGATGGTGTATGAAAATGCGAGTCGTTGGTTCGCTGAAGACGGTCAGTTGACCAAACGAATCTGCGAGGACACGTCTCTGTCCATCCCCGACTGCTGCGCCTGCGACGACGCTAAATATAGGATGGTGTTCGAGGGGCTCTGGTCTCCGCAGACACATCCAAAGAACTTCCCGACCCAAGCTCTGTGGCTAACTCACTTCTCTGACGTCATCGGAGCCACTCATCCTAAGAATTTCACTTTCTGGGGCGAAGGACAGCTCGCTTCAGACGGATTTAGATCTCTGGCGGAATGGGGTTCAGTAGGTCTGATGGAGCGTGAACTTCGTCAACACGGAGGTCTGCTCCGGTCCATCGTCAAGGCCCAGGGTCTGTGGCATCCGAGGGTCAATTCCAATACTTCTGCTGCCTTCACTGTTGACAAAAAGAGGCATTACCTCTCTCTTGCTTCTATGTTTGGTCCATCTCCTGACTGGGTGGTGGGAGTGAGTGGACTGGACCTCTGCCAGAAAGATTGCTCCTGGGTTGAATCTAAGATTATCGACTTGTATCCTTACGACGCTGGCACAGACAATGGCGTCTCTTACATGTCGCCCAATTCTGAGACCGTACCCCGGGAAAGGATGTACCGCATCACCCCCATGTTCCCCGAGGACCCTCGTGCTCCGTTCTACGACCCCGACTCCAAGACGATGGCTCCGATGGCCAGACTCTACCTCACCAGAGAAAAATTAATTTCCAAATCTTGTGACGAGGAAACTCTTTTAGCTCTCGTCGCTGAAGAGGAAGAGAACACGCAGACGGTCGATAAACCGCAATGTGCGGTGACGGAGTGGAGCGCGTGGTCGGAGTGCTCCGTGAGCTGCGGCAAGGGGCTCCGCATGAGGACGCGCCAGTACCGCCTGCCCGACAAGGCGCGCATGTTCTCCTGCGACCGCCAGCTCGTCTCCAAGGAGATGTGCGTCGCGCCCGTCGCCGAGTGCGACGGTGACGGCGAAGGTGACTCGGAGGCCGACAGCGTGGGCACCCCGGTGGAGGACCTGGACGGAGTGTGCAAGACCTACGACTGGGGCTCGTGGAGCGAGTGCTCCGTCACGTGCGGCGTCGGCATCAGCACCAGGAGACGACAGTTCGTCAACCACATGGGACTGAAGAAATGTCCGCTTGTGCAGATCGAAGAGAACCGCAAGTGCATGGAGCCGGCGTGCGCGGAGGAGGAGGCGGCGGCGGCGGCGGACCCGCGCTGCCCCACGTCGGCGTGGTCGGGCTGGTCGCCGTGCTCGGCGTCGTGCGGGCGCGGGGTGCGCTTCCGCACGCGGCTGCTGCTGGTGCCGGCCGACCAGCAGGCCGCGTGCGCCGCCGCCGTGGAGCTGCTGCAGCAGCGGCCCTGCTCCGACCGGGACGACTGCACCATCGACATGCTCACCGCCAAACGCATATGTATGGAGGAACCCACTCAAGGGCCGTGCAGAGGACTGTACCAGCGCTGGGCCTTCGTGGCCATGAAGGGGATGTGCATTCCCTTCAACTACGGCGGATGTCGCGGCAGCCAGAACAACTTCCTCACGCAAGAAGACTGTATGAACACTTGCAAGATTATGCTCGGTGGAGTAGTGCCGGGGGGATCAGTGCCTTCGCCGGCACTTCTACCGTCGCCCGTCGTCAGTTCCAACTATCCAGGACTCAGTATCAGCTCCATTGTGCCTCTTTCTAACGGTGTTGGTGGTCCTACCCTGACGGGAAGTCCAGGAGACTGTCAGGTGAGCCAGTGGAGCGACTGGAGCAAGTGCAGCCAGCCCTGCGGAGTCGGCTACCAGGAGCGCGTCAGGACTATTCTTGCACAGCCCGGCCCGGGAGGAGTGCCGTGTCCGAAGCTAATGTCGCGGCGCCGACGGTGCTTCAGGCGGTGTTAAGTACACACACAAATAAATGAAAATTATTATTTAGATTATGAACTTACCAGAATTTTATTTTTTATTTTTCTTAAGATTTATATTTTATGTTAAGACACTAATTATTATAGTGTAATCGTGTCTACTTAATCGAAATATTTTCTATGCTTTTTGTATTGTTTCCGATAAATACAAAATAATACGCAAAAAAAAAAAAAAAAA

>BmSPI49

ATGTCGTTCAGTTGGATCGTAGTGTTAGCGTTCGTCAACATCATCGTCCTCTGTACAGCTACTTGTCCAGAAAACGAAGAACGCACCTGCCTTCAAGGTTTATGCAGACCTCAGAAGTGCATCGAAAAAAATGATATCATCTTCTGCCAATTAGTTGATGAGGAGAAGTGCGAATATGGATGTGCCTGCAAAATAGGATATCTGAGAGACGAAAATGGAACTTGTATACCGCAAGACAAGTGTCCAACTGTGCCTTGTCCAGTGAATGAATACTTCACGAACTGCGCTAAGGGCATGTGTCGTCAGGAGAACTGCACAGAGCTGGGCAAGTTGTCTGAATGTAAAACGCAATCGACAGAGCTGTGCGAGCCGGGCTGCGTTTGTGAAGGGGGCTTCTTGAGATCAAAAAACGGAACGTGCGTATCTATCGATGAATGCCATAGGGAACTATGTCCCGTGAACGAAGTGTACTCGAGCTGTCGACAGCCCAATTGTAATTCTGATAAATGCGAGTACAAATACAGGTCACAGTCGTGTCCCTCGGACGAACCCTGCGAAGTCGGTTGTGTTTGTAAAAGAGGATTTCGTAGGGCTGACAATGGCACCTGTGTCGATGAAAGAGATTGTGAATCCCAGCTTTGTTCAGTAAATGAACAGTATTTAAGTTGCATCCAAGCTGTTTGTCGGGTCGAGAAGTGTTCAGACCTGGGGGGATCCCTCAGTTGCAAGGGGGTGTCGGAAAGGGAATGCGTCGGTGGCTGCGTCTGTAAGGACAACTACTTCCGAGCTAAAAACGACACTTGTATTAAACTCAGTGATTGTGACGCTGACCTTTGCTCTGAAAATGAAATACACGTGAACTGTGTCCTAGCACAGTGTGGCCCAATGACGTGCTCAGAGAAGGACGGTCCTATGTACTGCCCATCGGTGGACCAAAAGTCCTGCAAAGCCGGATGCGTGTGTAAAGAGGGATATCTAAAAGACGATAGCGGTAAATGTGTCGCCCGAGAAAATTGCCCAAATTAGGAATGTTCAGGCGAAAATGAAGAGTTTACCAACTGCACGAACCCATGTCCGCCGCGTACTTGCAATTCACTCGTCGCTCGTTTTGATTGTAGTAAGCCCAAACCCTGCGAAGAGGGATGCGCTTGCAAACCAGACTACTTGAAACTCGACGACAATTCGGCCTGTGTTAAGATTTGCGAATGTCCGCAAATGGCTAGCTCTCCTGACTGTCCAAAATTATAATGCAAAGTTAAATTAAAGATAATATGGATTTCAAAAA

>BmSPI48

TTGATTTTTTTTGAATATCAGCCGCGATGTCGTTTAGTTGGATCGTAGTGTTAGCGTTCGTCAACATCATCGTCCTCTGTACAGCTGACCTCTGCTCTGAAAATGAAATATACGTGAAATGTGTCCAAGCACATTGCGGCCCAAGGACGTGCTCAGAGAAGGACTTACCTATGCCCTGCCCATTGGTGAGGCAAGAGTACTGCAAAGCCGGATGCCTGTGTAAAGAGGGATATCTGAAAGACGATAGCGGTAAATGTGTCGCCCGAGAAAACTGCCCAAATTCTGACCTCTGCTCTGAAAATGAAATATACGTGAAATGTGTCCAAGCACAGTGCAGCCCAATGACGTGCTCAAAGAAGGACGGTCCTAAGATCTGCCCATTGGTGGAGGAAAAGTCCTGCAAAGCCGGATGCGTGTGTAAAGAGGGATATCTGAAAGACGATAGCGGTAAATGTGTCGCCCGAGAAAACTGCCCAAATTCTGACCTCTGCTCTGAAAATGAAATATATGTGAACTGTGTCCAAGCACAGTGCAGCCCAATGACGTGCTCAAAGAAGGACGGTCCTAAGATCTGCCCATTGGTGGAGGAAAAGTCCTGCAAAGCCGGATGCGTGTGTAAAGAGGGATATCTGAAAGACGATAGCGGTAAATGTGTCGCCCGAGAAAACTGCCCAAATTCTGACCTCTGCTCTGAAAATGAAATATACGTGAAATGTGTCCAAGCACATTGCGGCCCAAGGACGTGCTCAGAGAAGGACTTACCTATGCCCTGCCCATTGGTGAGGCAAGAGTACTGCAAAGCCGGATGCCTGTGTAAAGAGGGATATCTGAAAGACGATAGCGGTAAATGTGTCGCCCGAGAAAACTGCCCAAATTAGGAATGTTCAGGCGAAAATGAAGAGTTTTCCAACTGCACGAACCCATGTCCGCCGCGTACTTGCAATTCACTCATCGCTCGTATTAAGTAGTAAGCCCAAACCCTGCGAAGAGGGATGCACTTGCAAACCAGATTACTTGAAACTCGACGACAATTCGGCCTGTGTTAAGATTTGCGAATGTCCGCAAATGGCTAGCTCGCCTGACTGTCCAAAATTGTAATACAAAACAAAAAAATAATAATAATAGCATGGCTTTCAAAAACATTATTCTATATTTTTTCAAGTTAATTAAGAAATTATTCTCATATAAAAGTGCTCTAAAATATTGTAAAACTCATCATAGGTAAACAAAAATATTTATTTGTTATTAAATTGACAAATTATTATTAAAACAAAAAAAAAAAAAAAAAAAAAAAAAAAAAAAAAAAAAAA

>BmSPI45

ATTCTGTTTTATATCTACGGTTGCAGCATATTATGTACAAGAGCATCATTTATTACATCGTTGTCCGAGAAACGAACATTTCGTTGGAAACAGCACCGACTGCCCGATAACATGTTCGAACCGTTACCAGACAGTGCCGGGAGATTCGTGTGAAACTTACTCGGGATGCGTATGCGATGAAGATCGGGTTCGGCTATTCGATGATGCCACTGGACCTTGCGTTTCGACGGATTCGTGTCCGCCAGATCCAGTTTACTGTGGGCCACACGAAGAACCGACAGAATGTGAGAATATCTGCTTAGAGTCCTGCGCTCATGTTTATTCTAACGAAATCTGCATCGCCAGTAACCTTTGCATCCCTGGATGCAAATGTACTAAAAACCACTTGAGAAACCAATTGGGAATGTGCGTTCCGCGTGATTACTGCCCAGATTCGAACATCTGCTCGCCAACGTGCGCTCAACCTAATCCACCGGACTGTCCTAATATTGATCGTAATGTATGCGAACCTGGTTATATATTGTCTGAGATCGGAGGCGTCTGCATAAAGATCGAAGATTGTCCTGCTGATGCATCCTGCAATTCTGACCCGAATGCTATAATCGCTCAGTGCCCACAACCGTGTCCTTCGACGTGTGAAGCCCCTAATGCTGTTCCGTGCAAGAAAATGTGCGAACCCGTCGGATGTGAATGCAAGCCCGGCTTCATCCGGTCAAAGGTCAACGGCAAATGCATTTTACTTGATCAGTGTCCTGGAGGCAATCCCTGTGGAGACAACGCGACTTTCATGAATTGTCGGGTGCCTTGCATAACAGACTATTGTCCCGTTAACGACACCAGAGGCGAGGTCATTTGCGATATACCCAACCCTTGCCTTTCTGGATGTGTTTGTAACTCCTACTATAAGCACCGAAGCGTCAATGACAATCAATGTATCCCCGCCAAAGAGTGTCCACCGGTCAAGTGCACCAGACCGAATGAAGTCTGGGACTCTTGTCCATCGACGTGCCTATACGAAAATTGCAACGATGTGGACAATCCGAATGTAGTGTGCGACGACAGTTGCAAAGCCGAACCCCGTTGCGTGTGCGACGAGAATCATTTTAGAAACAACGACGGAGTGTGTGTGCCAGCTGAAGAATGTCCTTCCTACGTCATCAATACTGAACGCTGATGGAAGACGAATGGCATTTGTAAAAATTGTAACTATTAAAAAATATTAATAATAAAAGAAATATAAAAAAAAAAAA

>BmSPI43

GGCATAATGTCGTCCAAGAGCATATTTATCTGTCTATCATTATGTTATATCGTTAACGAAGTCTACGGTCAAGTAGCCACATTGTGTAGAGCAAACGAGCGTTTTTTGGAATGCGGCTGCCGTAAAACGTGTCGGAACCCAGCGCCGAACTGCAGAGCGATGTGCATTACCGGCTGCTTCTGCGAGGAGGGACAAGTTAACAACGACAACGGGGTCTGCGTGAACTTAGCCGACTGCCCTCAAGCCGCTTCCGCATACAAGCTGCAAACAACAGAGCCACGCTTCGATGGAGGAAAGTGCCCTCAAAACGAGGAGTACAAATTCTGCGAGCCCTGTAACAGGACTTGCGAGAATCCGTTCCCGGTGTGTCCAGCGCAGTGCGCCCGAGGATGTTTCTGTAAAGACGGTCTGGTGAGGGACAAAGACGGAAAATGTGTGGAATTGGAACAGTGCTCAAACTTAAAACATCTTAAGCTTGGAGAAAACTACAAACAGCCAATCCCTTCCCGCGTCAACTGCGGTCCCTATCAAGTGTACAAGAAGTGTGGCACCTGTGACAAAACCTGCAGTAATCCTAACCCTGTGTGCAACCAAACCTGTCAGAGAGGATGCTTCTGCCAAGAGGGCTATGTTAAATCTGTTCACGGAAGCTGCGTGAAACCGGAAGATTGTCCTGATGGTTCGTAGACGGAGCAATATGTTCGATATTCGCAAACACTGGTATTCTTAAACTTCTTTTCATTAATAGAATTTTTAGTATTTTTCTGAATCGATATAAAACAAATGGCGTTCTTATTTAGTCTCTACTTATATTATCTCTGTATCAAAAA

>BmSPI40

GTCAAGATGGCCGCCAAACATAACTTCCCCATGCTCCTGCTCGTATCCCTGATGGCTCTCGCAGCCAGCCAGGGTTCGGTTGAAAAAGATTGTCCTGAGAATTCTCATTTGACAATGAACCCCTGCGCGCCGACCTGTGAAGATCCAGACCTGACACACACCAGCTGCGTAGCGGCATTGCTCCCAACATGCCACTGTGATGATGGCTTCTTGTTCGACAAATCTGGAAAATGCGTACCCGTTGATGAATGTCCAGACCAAGAGAGTGACTGTATCAACTGATTCCGACTTATAATAGGCAGCGGCTTGGCTCTGCTCCTGGCATTGCTGAAGTCCATGGGCGACGGTAACCACTTACCATCAGGTGGGCCGTATGCTCGTCTGCCTAAAAGGGCAATAAAGATAATAAAATAATAATAA

>BmSPI38

ATAGTTATTGGAAAGCTGTAGTCTGAAAAATATCACGAAGATGGCCGCCAAACAATACTTTATCGTGTTCCTGATTGTCGCCGTGATGGCTCTCGAGGCCAGCACTACCGAATATGGATGCCCTGAAAATGCCCATTGGACTGATGACCCCTGCGTGAGGACCTGCGACGACCCGTACCTGACTAACACCGCTTGCGTCGGCGCCCTCATTCAAACCTGCCACTGTAACGACGGCTTAGTCTTCAACGCGGACAGAAAATGTGTGCCCATTTCTGATTGTTAAAATGTCATAACGATTTATAATCTTTTTTCGTGATTTTATTAAATGTTCAAATTGAAAAAAAAAAAAAAAAAA

>BmSPI36

AGTTCGCAACACATCGTTAAGATGGCCGCCAAACATTACTTCATCATGTTTCTGCTCGTGTCTCTGATGGCTCTCGCAGCCAGTAAGAGCTTGTTCGAGAAATCGTGTCCTGAAAATGCTCACACGACATTGAACCCCTGCGTGCCGACCTGTGCAGACCCGGAATTGAAACACACCAGCTGCGTGACGGCTTTCATCGCAACCTGCCACTGTGATTCTGGATACCTCTTCAACTCGGAGGGAAAATGCGTGCCCGTTGCCGAATGTTGAATATGACGCGGAGGCTATTTCAACTGATTTCGATTTGTATTTTATAAAAAAAGTTTATCTAACTATAACTAAACTATACTTGAGACCTTAGAACTTATATCTCAAGGCGCATTTACGTTGTAAATGGCTATGGGCTCCAGTAACTATTTAACACCAAGTGGGCTGTTAGCTCGTCCATACATCTAAGCAATAAAACATATATCCAAAAATTGAAGTTAAATATAGATACATTTAAGCTTACATATCTGCTGTTTTTAATAAAAAAAAGAAATAAAAGAAAAAAAAAAAAAAAA

>BmSPI35

GATAGTTATTCGAGAGTTGTAGTTTGAAAAAGATACTGAGATACTTACGAAGATGGCCGCCAAACAATACTTTATCGTGTTCCTGATTGTCGCCCTGATGACTCTTGGTGCCAGCACTGCTGACGATGGGTGTCCCGAGAATGCTCATTGGACTGATGACCCCTGCGCGAAGACCTGCGACGACCCATACTTGACTAACACCGTTTGCATCGCCGCCCTCATTCCAACCTGCCACTGTAACAGCGGCTTAGTCTTCAACGCGGAGAGAAAATGTGTACCCATTTCGGATTGTTAAACGATGTCACAACGATTTATAGCCTTATTCGTGATTTTATTAAATACTAGATGATTTTTGATAACGCCATCTGTTGTGTCTTTAAAGCAGTTAGTTGCTCTCAATTAAGAAAAATAGTCTATTATTATTATTCGCCAATAGATGTCGGGAAGAGTCATAAAGTTGATTATCGATAACACGAATAAAACAACATTTTCTGAAAATAAATCGTAGCTAGATCGAATTATCGCCCCCGAAATCCCCTGTATACTAAATTTTATGAAAATCGTTGGAGCCGTTTTAGAGATTCAGATTATATAAGTACCTATATATTAATTTACAAGAATTTCTCGTTTAAAGATAATTGCTCGATAATAATAATAATAATAAGATTCAAAAACAAAAAAAAAAAAAAAAAAAAAAAA
