# Supplementary figures and images for: Genome-Wide Identification and Immune Response Analysis of Serine Protease Inhibitor Genes in the Silkworm, Bombyx mori
Source: PLoS One. 2012 Feb 13;7(2):e31168. doi: 10.1371/journal.pone.0031168 (PMC3278429; doi:10.1371/journal.pone.0031168)

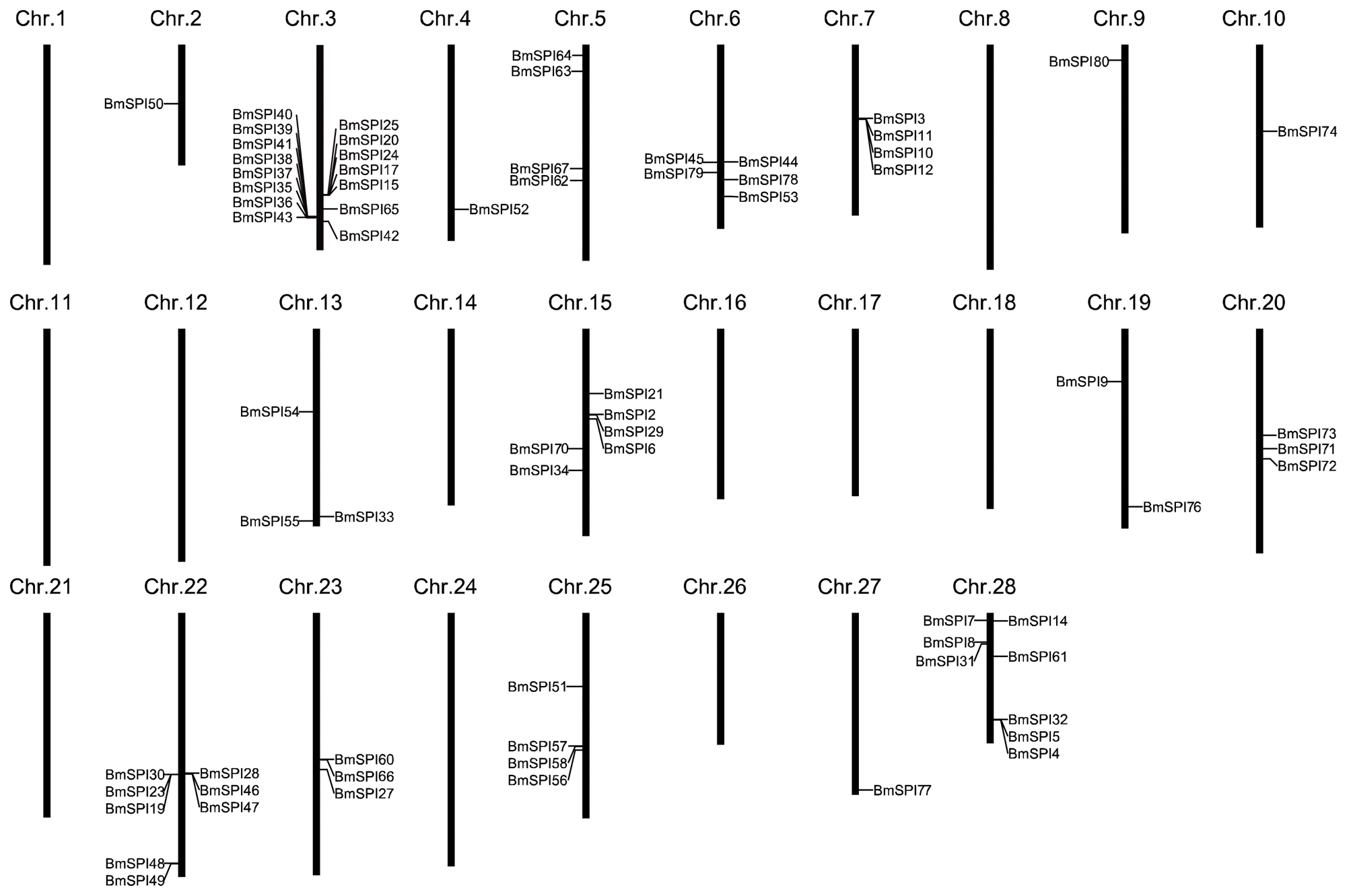

Supplement: Figure S2 — The chromosomal distribution of serine protease inhibitor genes in B.mori . (TIF) [file pone.0031168.s002.tif]

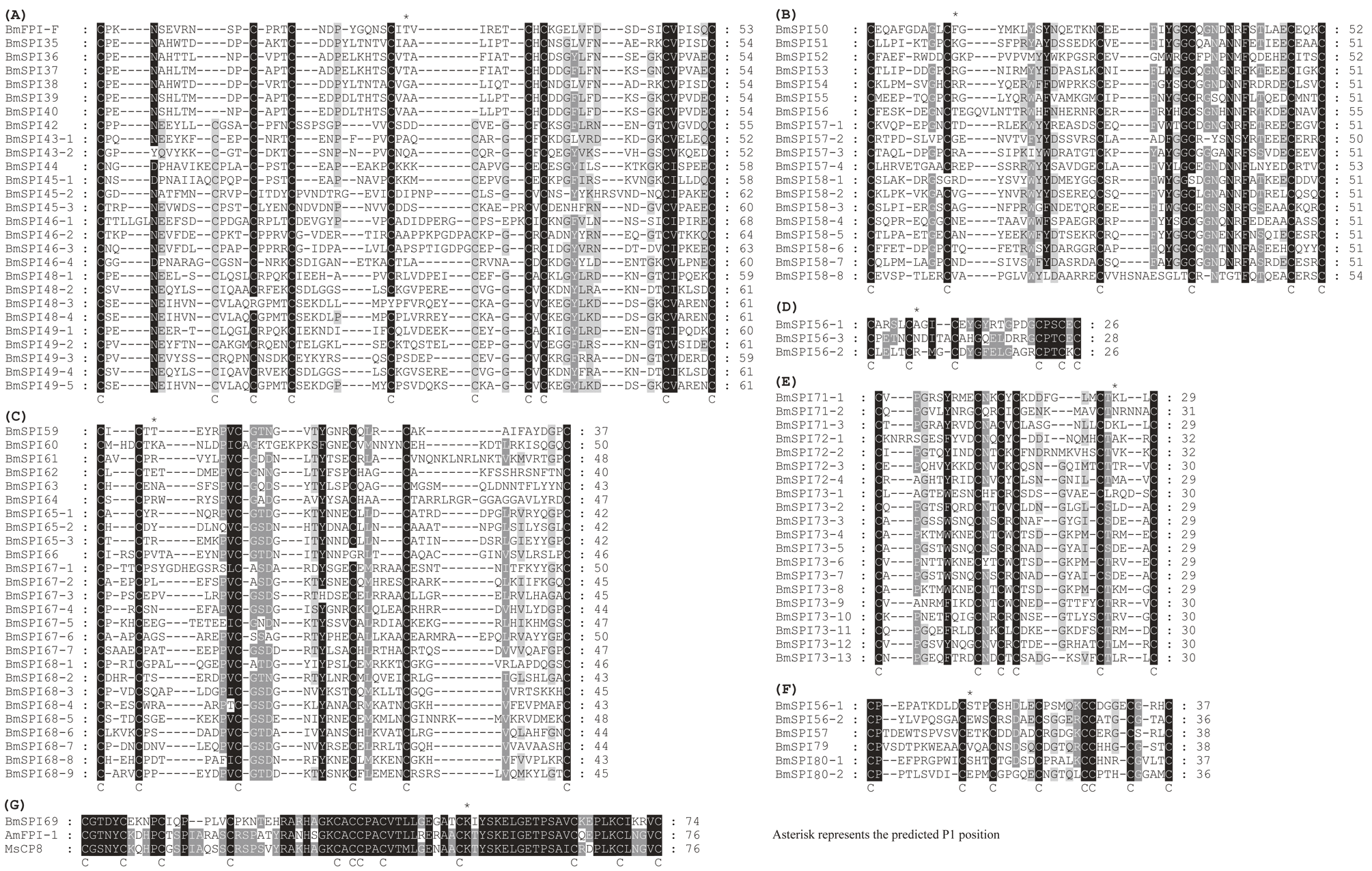

Supplement: Figure S3 — Sequence alignments of serine protease inhibitor domains. Alignments were done using ClustalX 1.83 with default parameters and shading was done using GENEDOC. Identical residues are shaded black, while similar residues are gray. Asterisk represents the predicted P1 position. (A)TIL, (B) Kunitz_BPTI, (C) Kazal, (D) Antistasin, (E) Pacifastin, (F) WAP, (G) amfpi. (TIF) [file pone.0031168.s003.tif]
