# Supplementary material for: Genome-Wide Identification and Immune Response Analysis of Serine Protease Inhibitor Genes in the Silkworm, Bombyx mori
Source: PLoS One. 2012 Feb 13;7(2):e31168. doi: 10.1371/journal.pone.0031168 (PMC3278429; doi:10.1371/journal.pone.0031168)
Supplement: Table S1 — The serine protease inhibitors in the silkworm. (PDF) [file pone.0031168.s005.pdf]

Supporting Information Table 1

The serine protease inhibitors in the silkworm

Name	Name in silkDB	SPI domain	Numbers of SPI domain	Chr.#	Scaffold #	Starting point	End point	Chain	EST	Annotation
BmSPI1	BGIBMGA009953	Serpin	1	/	nscaf2979	113003	118777	-	Yes	Serpin1
BmSPI2	BGIBMGA007720	Serpin	1	15	nscaf2888	1695601	1703011	-	Yes	Serpin2
BmSPI3	BGIBMGA010212	Serpin	1	7	nscaf2986	2025913	2049705	+	Yes	Serpin3
BmSPI4	BGIBMGA013852	Serpin	1	28	nscaf3098	198532	199764	+	Yes	Serpin4
BmSPI5	BGIBMGA013849	Serpin	1	28	nscaf3098	156075	157103	+	Yes	Serpin5
BmSPI6	BGIBMGA007729	Serpin	1	15	nscaf2888	1281387	1287794	-	Yes	Serpin6
BmSPI7	BGIBMGA013903	Serpin	1	28	nscaf3099	534661	535860	-	Yes	Serpin7
BmSPI8	BGIBMGA013904	Serpin	1	28	nscaf3099	2672749	2675709	-	Yes	Serpin8
BmSPI9	BGIBMGA001983	Serpin	1	19	nscaf2204	4280050	4285863	+	Yes	Serpin9
BmSPI10	BGIBMGA010214	Serpin	1	7	nscaf2986	2083047	2086003	+	Yes	Serpin10
BmSPI11	BGIBMGA010213	Serpin	1	7	nscaf2986	2059013	2064447	+	Yes	Serpin11
BmSPI12	BGIBMGA010216	Serpin	1	7	nscaf2986	2098064	2104805	+	Yes	Serpin12
BmSPI13	BGIBMGA002368	Serpin	1	/	nscaf2439	2860	4292	+	Yes	Serpin13
BmSPI14	BGIBMGA013958	Serpin	1	28	nscaf3099	567124	568323	+	Yes	Serpin14
BmSPI15	BGIBMGA008831	Serpin	1	3	nscaf2925	475942	483150	+	Yes	Serpin15
BmSPI16	BGIBMGA003292	Serpin	1	/	nscaf2641	21922	28230	+	Yes	Serpin16
BmSPI17	BGIBMGA008830	Serpin	1	3	nscaf2925	461290	468785	+	Yes	Serpin17
BmSPI18		Serpin	1	/	nscaf2641	1197	7586	+	Yes	Serpin18
BmSPI19	BGIBMGA004735	Serpin	1	22	nscaf2814	364955	383519	+	Yes	Serpin19
BmSPI20	BGIBMGA008827	Serpin	1	3	nscaf2925	436242	442612	+	Yes	Serpin20
BmSPI21		Serpin	1	15	nscaf2888	3730190	3742067	-	Yes	Serpin21
BmSPI22		Serpin	1	/	scaffold2278	254	2426	-	Yes	Serpin22
BmSPI23		Serpin	1	22	nscaf2814	377650	383519	+	Yes	Serpin23
BmSPI24	BGIBMGA008828	Serpin	1	3	nscaf2925	445169	447682	+	Yes	Serpin24
BmSPI25	BGIBMGA008826	Serpin	1	3	nscaf2925	383697	388498	+	Yes	Serpin25
BmSPI26		Serpin	1	/	scaffold706	32832	37928	-	No	Serpin26
BmSPI27	BGIBMGA011587	Serpin	1	23	nscaf3027	4608138	4613908	+	Yes	Serpin27

BmSPI28	BGIBMGA004726	Serpin	1	22	nscaf2814	504860	511103	-	Yes	Serpin28
BmSPI29	BGIBMGA007721	Serpin	1	15	nscaf2888	1649315	1655134	-	Yes	Serpin29
BmSPI30	BGIBMGA004736	Serpin	1	22	nscaf2814	387723	398842	+	No	Serpin30
BmSPI31	BGIBMGA013903	Serpin	1	28	nscaf3099	2677905	2680242	-	Yes	Serpin31
BmSPI32	BGIBMGA013848	Serpin	1	28	nscaf3098	134013	135200	+	Yes	Serpin32
BmSPI33	BGIBMGA001066	Serpin	1	13	nscaf1898	703219	705634	-	Yes	Serpin33
BmSPI34	BGIBMGA003349	Serpin	1	15	nscaf2655	1012273	1016336	-	Yes	Serpin34
BmSPI35	BGIBMGA009072	TIL	1	3	nscaf2931	936942	937907	-	Yes	
BmSPI36	BGIBMGA009095	TIL	1	3	nscaf2931	942071	944291	+	Yes	
BmSPI37	BGIBMGA009073	TIL	1	3	nscaf2931	919347	920110	-	No	
BmSPI38	BGIBMGA009094	TIL	1	3	nscaf2931	912977	913700	+	Yes	
BmSPI39	BGIBMGA009092	TIL	1	3	nscaf2931	894015	896376	+	No	
BmSPI40	BGIBMGA009091	TIL	1	3	nscaf2931	881660	883488	+	Yes	
BmSPI41	BGIBMGA009093	TIL	1	3	nscaf2931	899792	899959	+	No	
BmSPI42	BGIBMGA009075	TIL	1	3	nscaf2931	551264	552975	-	Yes	
BmSPI43	BGIBMGA009096	TIL	3	3	nscaf2931	958549	972519	+	Yes	
BmSPI44	BGIBMGA006251	TIL	1	6	nscaf2851	1060348	1063762	+	No	
BmSPI45	BGIBMGA006235	TIL	3	6	nscaf2851	1089671	1100598	-	Yes	
BmSPI46	BGIBMGA004727	TIL	4	22	nscaf2814	486479	490449	-	No	
BmSPI47	BGIBMGA004728	TIL	6	22	nscaf2814	421025	435042	-	Yes	
BmSPI48	BGIBMGA010892	TIL	4	22	nscaf3005	1142843	1143684	+	Yes	
BmSPI49	BGIBMGA010889	TIL	5	22	nscaf3005	1095501	1100551	+	Yes	
BmSPI50		Kunitz_BPTI	1	2	nscaf2964	2742907	2744750	+	Yes	AAO17293
BmSPI51		Kunitz_BPTI	1	/	nscaf2818	112890	115513	+	Yes	AAL83944
BmSPI52	BGIBMGA003074	Kunitz_BPTI	1	4	nscaf2589	735178	735988	-	No	
BmSPI53	BGIBMGA006340	Kunitz_BPTI	1	6	nscaf2852	2595227	2596475	+	No	
BmSPI54	BGIBMGA001246	Kunitz_BPTI	1	13	nscaf1898	11070169	11076436	+	Yes	
BmSPI55	BGIBMGA001091	Kunitz_BPTI	1	13	nscaf1898	278132	291974	+	Yes	
BmSPI56	BGIBMGA005119	Kunitz_BPTI/ Antistasin/WAP	1/3/2	25	nscaf2823	1469785	1490431	+	Yes	
BmSPI57	BGIBMGA005130	Kunitz_BPTI/ WAP	4 /1	25	nscaf2823	1859028	1878038	+	Yes	

BmSPI58	BGIBMGA005129	Kunitz_BPTI	8	25	nscaf2823	1825365	1858352	+	Yes	
BmSPI59		Kazal	1	20	scaffold826	5493	7786	-	Yes	NP_001037047
BmSPI60	BGIBMGA011573	Kazal	1	23	nscaf3027	3578987	3579235	+	Yes	NP_001040250
BmSPI61	BGIBMGA014012	Kazal	1	28	nscaf3099	4075266	4077084	+	No	
BmSPI62	BGIBMGA003667	Kazal	1	5	nscaf2674	3633185	3648429	+	Yes	
BmSPI63	BGIBMGA002723	Kazal	1	5	nscaf2529	5168618	5181244	-	Yes	
BmSPI64	BGIBMGA013485	Kazal	1	5	nscaf3075	827184	849062	-	No	
BmSPI65	BGIBMGA009047	Kazal	3	3	nscaf2931	1782179	1783723	-	Yes	
BmSPI66	BGIBMGA011574	Kazal	1	23	nscaf3027	3614163	3615180	+	Yes	NP_001040294
BmSPI67	BGIBMGA003498	Kazal	7	5	nscaf2674	5095535	5109274	-	No	
BmSPI68	BGIBMGA013655	Kazal	9	/	nscaf3087	33689	46217	-	Yes	
BmSPI69	BGIBMGA008205	amfpi	1	/	nscaf2893	64178	65058	+	Yes	AY655143
BmSPI70	BGIBMGA007558	Kunitz (ITI)	/	15	nscaf2887	351002	376921	+	Yes	
BmSPI71	BGIBMGA004225	Pacifastin	2	20	nscaf2780	11962	16806	0	No	
BmSPI72	BGIBMGA004226	Pacifastin	4	20	nscaf2780	34504	35673	+	No	
BmSPI73		Pacifastin	13/11	20	nscaf2779-2800	(nscaf2779) 204740	(nscaf2780) 8369	+	Yes	Breugelmans et al., 2009
BmSPI74	BGIBMGA006856	Bowman-Birk	11	10	nscaf2859	732807	824065	+	Yes	
BmSPI75	BGIBMGA012631	α-macroglobulin	1	/	nscaf3053	468531	493450	-	No	
BmSPI76	BGIBMGA004193	α-macroglobulin	1	19	nscaf2770	1074754	1098181	+	No	
BmSPI77	BGIBMGA004536	α-macroglobulin	1	27	nscaf2797	1139344	1159911	+	No	
BmSPI78	BGIBMGA006306	α-macroglobulin	1	6	nscaf2852	943084	952132	+	Yes	
BmSPI79	BGIBMGA006297	WAP	1	6	nscaf2852	357420	376290	+	Yes	
BmSPI80	BGIBMGA008016	WAP	2	9	nscaf2889	1221751	1224798	-	Yes	
