# Supplementary material for: Genome-Wide Identification and Immune Response Analysis of Serine Protease Inhibitor Genes in the Silkworm, Bombyx mori
Source: PLoS One. 2012 Feb 13;7(2):e31168. doi: 10.1371/journal.pone.0031168 (PMC3278429; doi:10.1371/journal.pone.0031168)
Supplement: Table S2 — Multiple tissues expression data of SPI genes. (PDF) [file pone.0031168.s006.pdf]

Supporting Information Table 2

Multiple tissues expression data of SPI genes

Tissue	BmSPI74	BmSPI76	BmSPI56	BmSPI27	BmSPI10	BmSPI25	BmSPI6	BmSPI20	BmSPI80	BmSPI70	BmSPI57	BmSPI55	BmSPI66	BmSPI65	BmSPI62	BmSPI64	BmSPI17
Testis-Cy3-1	-139	597	371	447	360	1432	478	127	6388	6015	1997	5854	1224	368	1820	493	-28
Testis-Cy5-2	-260	367	337	413	342	1504	467	72	7908	5998	1740	5601	1385	184	1248	504	-36
Testis-Cy3-6	-18	421	393	567	332	1151	860	194	4819	3981	1420	2867	707	257	1340	606	72
Testis-Cy5-5	0	444	228	585	461	869	569	227	3894	4821	1123	2025	1035	164	875	310	112
Testis-Cy3-4	18	746	324	1003	345	2012	4393	7114	4446	5888	5257	2249	1670	269	1596	745	82
Testis-Cy5-3	-19	621	281	585	196	1311	2934	4748	4037	4521	4085	1657	1538	149	985	537	114
Ovary-Cy5-1	-41	326	187	684	181	700	217	2029	4159	5541	10596	20727	3023	1344	369	-13	-28
Ovary-Cy3-2	-110	80	284	604	224	739	291	1945	4747	6095	11182	23837	4549	954	334	-36	-32
Ovary-Cy3-5	21	252	299	815	337	741	461	1467	3128	4296	11158	15436	3594	595	338	21	93
Ovary-Cy5-6	18	203	288	652	267	621	460	1461	3630	3627	8688	19902	2330	637	394	25	40
Ovary-Cy5-4	18	341	224	710	420	749	3115	14220	2705	2543	15278	16795	4230	451	617	21	34
Ovary-Cy3-3	30	364	329	661	372	705	3874	12736	3112	2578	15821	14867	5112	395	620	22	74
Head-F-Cy5-1	193	399	481	108	1254	622	960	96	334	4766	19174	3070	11191	3037	257	9	459
Head-F-Cy3-2	127	66	516	95	1970	527	766	158	346	5704	18740	2825	21391	3901	278	29	204
Head-M-Cy3-1	369	485	650	182	1789	997	1893	146	518	5411	18477	3874	13780	4186	351	32	553
Head-M-Cy5-2	186	127	678	124	2593	693	1437	204	350	8018	18374	4468	27979	4078	321	51	284
Integument-F-Cy5-1	745	311	408	67	3264	435	395	178	4961	8976	5290	14448	24904	845	574	7	52
Integument-M-Cy5-2	406	485	599	90	3482	620	852	206	2653	15382	10424	12944	45645	708	442	-30	171
Integument-F-Cy3-2	537	261	564	92	2682	652	434	150	2414	9165	6364	8620	35068	610	316	27	118
Integument-M-Cy3-1	790	534	744	166	4503	454	862	284	4683	16317	8290	17890	28963	818	605	45	167
Fat body-F-Cy5-1	-10	149	158	106	39	144	51	86	8020	12959	12486	15360	325	598	189	17	13
Fat body-M-Cy3-1	28	223	392	206	143	216	66	133	6206	17723	13271	19041	1146	415	329	64	128
Fat body-M-Cy5-2	-24	141	329	17	95	144	16	122	7232	14381	11040	20603	730	506	420	10	8
Fat body-F-Cy3-2	-25	232	330	73	113	208	82	231	9222	14454	14339	20266	492	1044	402	-3	18
Midgut-F-Cy5-1	-36	210	305	-32	63	445	284	4890	63	152	4995	17	210	2851	464	-1	213
Midgut-M-Cy3-1	23	230	417	113	115	403	138	3969	90	140	3222	104	367	2113	325	23	159
Midgut-M-Cy5-2	-50	291	623	-54	10	399	135	4238	88	105	4082	74	430	1964	277	44	182
Midgut-F-Cy3-2	7	322	810	68	108	627	309	4631	50	166	4883	42	556	2641	405	15	186
Hemocyte-F-Cy5-1	-7	172	181	5	5	346	1666	-14	81	99	27715	7343	3338	1056	114	14	19
Hemocyte-M-Cy3-1	10	182	166	29	12	363	1142	-31	77	112	36782	8965	5936	2404	128	8	12
Hemocyte-M-Cy5-2	-35	149	197	-1	-24	263	895	0	15	35	31778	10534	4198	2541	127	28	-14
Hemocyte-F-Cy3-2	-18	112	356	13	1	313	1301	-39	19	79	20486	7843	2255	1220	122	16	-28
Malpighian tubule-F-Cy5-1	-12	233	220	21	90	233	419	99	172	514	1175	380	241	272	332	-17	30
Malpighian tubule-M-Cy3-1	-19	223	372	0	78	524	732	259	85	610	1102	538	255	143	467	-9	22
Malpighian tubule-M-Cy5-2	35	447	414	27	159	755	266	447	137	881	1568	947	370	310	620	24	79
Malpighian tubule-F-Cy3-2	42	265	457	-19	92	272	215	46	165	834	1295	388	438	153	357	-15	42
Malpighian tubule-F-Cy5-3	-32	220	395	6	51	311	203	210	160	832	1210	823	424	703	620	23	15
Malpighian tubule-M-Cy3-3	39	320	458	25	105	654	229	599	176	925	1195	1309	492	566	865	20	32
Malpighian tubule-M-Cy5-4	0	469	190	40	241	702	325	503	135	819	1860	1208	347	457	922	19	61
Malpighian tubule-F-Cy3-4	97	439	292	200	302	519	374	291	249	996	2236	828	480	797	868	26	147
A/MSG-F-Cy5-1	-16	97	235	28	378	134	-62	-28	5	18	470	-23	85	72	160	23	99
A/MSG-M-Cy3-1	6	186	248	59	560	419	1	6	26	53	469	47	188	128	305	27	212

A/MSG-M-Cy5-2	-16	172	348	56	557	396	-7	8	13	34	370	38	169	91	237	28	197
A/MSG-F-Cy3-2	20	200	334	68	302	223	-25	24	37	49	535	26	223	94	211	56	135
A/MSG-M-Cy3-3	95	252	622	119	575	611	7	28	122	13	780	123	270	213	696	11	135
A/MSG-M-Cy5-4	44	271	396	49	599	641	24	29	70	97	756	37	138	103	608	0	236
A/MSG-F-Cy5-5	14	343	278	92	595	394	-5	105	7	28	893	88	194	125	447	-2	275
A/MSG-M-Cy3-5	41	324	351	57	522	507	-3	39	26	58	702	94	252	141	367	-22	320
A/MSG-M-Cy5-6	21	349	162	63	296	542	25	16	24	36	328	83	165	132	434	3241	234
A/MSG-F-Cy3-6	43	276	327	91	333	464	-20	18	54	62	527	-30	236	145	354	9603	5
A/MSG-F-Cy3-4	48	209	397	80	483	457	66	0	75	146	972	154	179	244	707	12	188
A/MSG-F-Cy5-3	-15	268	474	38	477	372	-23	4	36	88	866	190	196	245	676	21	31
PSG-F-Cy5-3	37	204	681	18	86	378	-25	-11	14	1	911	24	220	191	356	18	369
PSG-M-Cy5-4	62	354	586	52	55	563	8	-8	51	23	489	57	228	140	409	15	1156
PSG-F-Cy3-4	119	382	711	102	171	576	38	11	90	75	732	95	375	320	530	72	406
PSG-M-Cy3-1	15	197	368	17	43	240	7	24	32	60	527	74	161	145	337	41	597
PSG-M-Cy3-3	32	243	659	82	58	358	-12	74	24	21	549	47	254	231	349	57	1518
PSG-M-Cy5-2	-33	163	271	8	16	198	-21	-19	35	29	358	30	91	137	219	29	2165
PSG-F-Cy3-2	11	333	381	66	25	393	30	5	72	200	726	94	139	239	281	54	3118
PSG-F-Cy5-1	-5	209	350	39	50	277	-38	-4	16	129	767	169	157	136	209	16	397

Continue

Tissue	BmSPI12	BmSPI9	BmSPI5	BmSPI11	BmSPI4	BmSPI2	BmSPI16/18/22	BmSPI38	BmSPI36	BmSPI49	BmSPI45	BmSPI48	BmSPI39	BmSPI37	BmSPI46	BmSPI47
Testis-Cy3-1	223	11024	3174	1976	8453	3187	214	3050	1690	316	510	94	439	80	-26	-59
Testis-Cy5-2	139	10226	3170	2376	6721	3276	175	3768	1427	324	591	-43	324	30	-40	-154
Testis-Cy3-6	295	9790	1812	1902	6335	2446	331	2607	1300	282	536	175	400	345	167	21
Testis-Cy5-5	222	8757	1593	2007	5177	1972	221	2762	1307	277	553	137	207	147	60	6
Testis-Cy3-4	1068	4741	3436	2265	8125	2961	331	654	149	222	1204	93	336	189	97	38
Testis-Cy5-3	873	3078	2310	1790	6605	2183	173	604	107	174	903	41	215	95	116	2
Ovary-Cy5-1	660	43181	7295	4162	3440	3503	-31	14459	4532	653	306	298	19	174	68	6
Ovary-Cy3-2	954	41340	7892	4227	3174	3648	-13	16592	4444	801	368	325	8	148	89	-74
Ovary-Cy3-5	1177	33236	5315	4134	2948	2762	30	27876	6389	1584	421	1204	74	358	106	21
Ovary-Cy5-6	1342	31674	5831	4772	3403	3069	29	29325	5926	1551	342	1196	59	340	79	-1
Ovary-Cy5-4	2666	1337	5231	2875	3369	2461	15	3194	195	316	737	100	25	122	84	-3
Ovary-Cy3-3	2788	1338	4886	2997	3838	2696	39	3596	252	381	696	122	52	138	101	-5
Head-F-Cy5-1	3246	26391	21164	17800	7344	4292	10915	69207	15333	3586	16758	1578	570	700	69	-1
Head-F-Cy3-2	4379	40588	22819	22033	11972	4072	10602	101488	21750	5143	21091	2006	433	977	90	11
Head-M-Cy3-1	4127	44381	25574	20342	8445	4486	314	55851	12971	4193	1552	1981	236	870	99	-10
Head-M-Cy5-2	7076	72564	26421	38004	11811	5191	10	132551	28680	5099	642	2057	58	984	65	29
Integument-F-Cy5-1	5895	73973	31004	21729	6559	3451	911	40481	17336	6546	866	2490	128	573	55	31
Integument-M-Cy5-2	8572	140572	31403	28466	9029	5510	363	75026	23630	7287	713	3375	201	900	77	38
Integument-F-Cy3-2	5318	43540	24922	21528	7040	3830	1269	37931	20656	7318	841	3373	257	770	65	34
Integument-M-Cy3-1	7772	96172	33197	22926	7764	4776	409	62895	19806	7494	521	2910	195	742	89	108
Fat body-F-Cy5-1	5133	70092	10022	996	1186	3013	20	43947	14714	10899	537	4816	133	328	10	-8
Fat body-M-Cy3-1	5530	91934	8889	1858	1702	3335	150	34612	11183	10159	13899	3781	568	295	80	35
Fat body-M-Cy5-2	5324	56029	9217	1711	1585	2498	138	50252	10652	8578	10688	2744	409	297	53	7
Fat body-F-Cy3-2	6636	59464	15211	1455	1686	3786	94	54186	17236	14169	3982	4925	343	483	56	32

Midgut-F-Cy5-1	68	170	8630	576	519	8953	21	115782	320	359	368	26	59	131	11	5
Midgut-M-Cy3-1	119	1901	7640	535	585	8056	35	105424	491	396	319	81	104	197	37	81
Midgut-M-Cy5-2	88	2110	8103	467	450	8825	94	168432	647	413	691	57	57	243	-40	57
Midgut-F-Cy3-2	143	198	9401	571	491	9034	70	126848	531	541	836	136	143	329	22	17
Hemocyte-F-Cy5-1	4357	482	9280	10220	8739	2511	43	1439	129	146	206	3	4	111	-1	-13
Hemocyte-M-Cy3-1	6060	258	11301	15601	10285	2410	53	542	92	137	248	43	18	157	26	4
Hemocyte-M-Cy5-2	5997	101	8718	10139	9945	2484	24	468	122	93	254	24	-16	106	-8	-72
Hemocyte-F-Cy3-2	4341	149	7410	6350	9076	2659	46	1202	126	99	227	91	6	112	-3	0
Malpighian tubule-F-Cy5-1	3556	15093	4524	911	1227	1896	17	22706	2208	2402	1901	1058	246	142	34	-4
Malpighian tubule-M-Cy3-1	4266	9910	5058	770	1848	2465	20	3505	1286	3627	1568	1423	158	177	87	-17
Malpighian tubule-M-Cy5-2	7068	15239	5582	989	2491	3788	71	4777	1898	3903	398	1373	293	263	35	-35
Malpighian tubule-F-Cy3-2	3782	17797	4366	999	1291	3071	88	25873	2975	2160	2733	1026	518	161	19	-34
Malpighian tubule-F-Cy5-3	4667	16621	7948	623	1862	2181	49	25256	2640	2030	3018	658	172	241	28	-34
Malpighian tubule-M-Cy3-3	6173	10795	8059	513	2662	2484	88	5073	1565	3092	552	890	157	223	54	8
Malpighian tubule-M-Cy5-4	8023	11927	7832	1638	2162	3058	44	4886	1955	7360	744	1752	126	241	71	10
Malpighian tubule-F-Cy3-4	5325	18441	7218	1867	1693	2584	49	26405	3298	5189	4130	1532	292	534	105	111
A/MSG-F-Cy5-1	138	122	2164	1322	1144	1244	16163	60377	21870	4939	54316	764	1861	5590	561	1462
A/MSG-M-Cy3-1	341	662	2548	1906	2001	1485	30902	36113	15473	28319	36100	3509	3080	4559	2335	6707
A/MSG-M-Cy5-2	274	574	2288	1705	2073	1709	29275	50508	23166	21420	45042	2772	2528	4725	1998	5373
A/MSG-F-Cy3-2	172	167	2022	1507	1441	1686	16397	60834	20984	5687	47380	884	1649	4640	722	1539
A/MSG-M-Cy3-3	79	1203	3102	2601	1771	2346	39286	65038	28013	27273	65062	3949	5518	6522	3694	7837
A/MSG-M-Cy5-4	235	1254	3086	2230	1747	1762	47764	57546	24483	23719	57581	3407	6588	6025	3454	6406
A/MSG-F-Cy5-5	352	246	2154	1577	1627	1587	37984	64822	29674	13735	64691	1510	3500	4974	2436	3668
A/MSG-M-Cy3-5	342	798	2286	1745	2195	1750	38664	38925	19894	26143	38912	3228	3574	4344	3106	6642
A/MSG-M-Cy5-6	196	373	2047	729	1557	2619	46932	94509	23918	14973	42340	2916	2475	4752	1595	4370
A/MSG-F-Cy3-6	87	112	2049	779	1288	2619	35493	74776	23278	8811	36013	1850	2055	4617	1610	2496
A/MSG-F-Cy3-4	219	3067	2770	1317	1778	1642	41174	50493	16755	15800	47715	2697	5112	3798	3140	3163
A/MSG-F-Cy5-3	87	2540	2620	1334	1464	1939	48058	52154	26580	19639	52184	2362	5456	4725	3160	3320
PSG-F-Cy5-3	49	202	768	448	1234	1419	24770	44909	15885	3777	44947	910	474	1230	956	947
PSG-M-Cy5-4	3	622	975	194	1097	2412	10816	10428	6111	3794	65892	1370	378	408	642	1725
PSG-F-Cy3-4	104	307	1067	379	1080	2081	27607	61311	16103	3132	58414	1237	563	1247	790	1106
PSG-M-Cy3-1	36	789	814	354	2979	1357	10451	17181	6016	3435	27003	606	163	365	570	736
PSG-M-Cy3-3	32	631	723	358	1650	1535	8986	6872	4117	5812	34516	1399	395	421	1031	2075
PSG-M-Cy5-2	0	583	723	238	2232	1189	9345	12011	5898	2677	30466	576	108	425	432	495
PSG-F-Cy3-2	48	2987	741	303	1745	1353	885	3609	1979	1997	34461	509	129	254	642	490
PSG-F-Cy5-1	10	3314	713	248	2024	1379	818	5220	1964	1596	35282	298	70	172	543	406
