# Supplementary material for: Genome-Wide Identification and Immune Response Analysis of Serine Protease Inhibitor Genes in the Silkworm, Bombyx mori
Source: PLoS One. 2012 Feb 13;7(2):e31168. doi: 10.1371/journal.pone.0031168 (PMC3278429; doi:10.1371/journal.pone.0031168)
Supplement: Table S3 — Developmental expression data of SPI genes. (PDF) [file pone.0031168.s007.pdf]

Supporting Information Table 3

Developmental expression data of SPI genes

Developmental stage	BmSPI61	BmSPI62	BmSPI76	BmSPI72	BmSPI71	BmSPI52	BmSPI53	BmSPI56	BmSPI68	BmSPI27	BmSPI15	BmSPI25	BmSPI6	BmSPI10	BmSPI12	BmSPI9	BmSPI5	BmSPI11	BmSPI4	BmSPI2
day 3 of fifth instar(mix)	-88.166667	-88.1667	-88.1667	-88.1667	-88.1667	-88.1667	-88.1667	-88.1667	-88.1667	-88.1667	-88.1667	-88.1667	-88.1667	330.6667	2299.833	24849	10002.5	8567.5	3744.333	4069.667
day 4 of fifth instar(male)	-56	-56	-56	-56	-56	-56	-56	-56	-56	-56	-56	-56	-56	564	-56	22145	4487	6340	1629	2876
day 4 of fifth instar(male)	-73	-73	-73	-73	-73	-73	-73	-73	-73	-73	-73	-73	-73	775	-73	25069	2606	4804	1137	2763
day 5 of fifth instar(male)	-112	-112	-112	-112	-112	-112	-112	-112	-112	-112	-112	-112	-112	-112	1102	27556	5897	9475	2880	3052
day 5 of fifth instar(male)	-92	-92	-92	-92	-92	-92	-92	-92	-92	-92	-92	-92	-92	-92	1919	32246	4791	11434	3555	2284
day 6 of fifth instar(male)	-109	-109	-109	-109	-109	-109	-109	-109	-109	-109	-109	-109	-109	1352	2943	40927	5170	5168	3199	1932
day 6 of fifth instar(male)	-135	-135	-135	-135	-135	-135	-135	-135	-135	-135	-135	-135	-135	1330	3036	39437	6058	5159	3937	1780
day 7 of fifth instar(male)	-157	-157	-157	-157	-157	-157	-157	-157	-157	-157	-157	-157	-157	1417	1489	14691	6718	4875	3249	1949
day 7 of fifth instar(male)	-87	-87	-87	-87	-87	-87	-87	-87	-87	-87	-87	-87	-87	1593	1582	11890	5685	6421	3204	1433
start of wandering(male)	-116	-116	-116	-116	-116	-116	-116	-116	-116	-116	-116	-116	-116	1189	4273	31016	10028	8507	2893	1951
start of wandering(male)	-72	-72	-72	-72	-72	-72	-72	-72	-72	-72	-72	-72	-72	828	3354	30456	12276	8421	3556	2301
12h after wandering(male)	-64	-64	-64	-64	-64	-64	-64	-64	-64	-64	-64	611	5334	1181	2540	1498	13341	4342	4707	1096
12h after wandering(male)	-73	-73	-73	-73	-73	-73	-73	-73	-73	-73	-73	879	3982	1398	1950	1861	18639	3722	4792	1513
24h after wandering(male)	-37	389	-37	-37	-37	-37	-37	-37	-37	548	-37	660	3737	483	1261	1489	11221	5249	4590	924
24h after wandering(male)	-76	396	-76	-76	-76	-76	-76	-76	-76	683	-76	680	2255	527	1139	2024	14080	5044	3498	1074
36h after wandering(male)	-65	511	-65	-65	-65	-65	-65	-65	3146	2941	-65	862	3492	2096	735	2764	13699	12830	2193	1435
36h after wandering(male)	-105	718	-105	-105	-105	-105	-105	-105	3558	3180	-105	1109	3782	2493	657	2023	15279	7819	3055	1626
48h after wandering(male)	-52	-52	-52	-52	-52	-52	-52	-52	-52	-52	-52	972	13356	-52	1067	1983	11691	10111	4124	2109
48h after wandering(male)	-98	-98	-98	-98	-98	-98	-98	-98	-98	-98	-98	1168	13658	-98	1163	2335	12742	11137	4015	2076
60h after wandering(male)	-66	-66	478	-66	-66	-66	-66	-66	-66	466	-66	1471	3849	645	2923	3134	21593	17966	4816	2695
60h after wandering(male)	-54	-54	559	-54	-54	-54	-54	-54	-54	544	-54	1223	3920	485	2805	2318	13742	13306	5386	2781
72h after wandering(male)	-198	-198	-198	-198	-198	-198	-198	-198	-198	-198	-198	-198	-198	-198	1232	4343	9463	3452	3345	1424
72h after wandering(male)	-126	-126	-126	-126	-126	-126	-126	-126	-126	-126	-126	-126	-126	-126	1312	3713	10023	3746	4157	1453
96h after wandering(male)	-91	-91	-91	-91	-91	-91	-91	-91	-91	917	-91	1169	1176	2187	1583	8448	8790	3347	3367	1580
96h after wandering(male)	-79	-79	-79	-79	-79	-79	-79	-79	-79	1032	-79	1358	1755	2360	1789	7102	8387	3802	3588	1332
120h after wandering(male)	-134	574	-134	-134	-134	-134	-134	-134	1174	7489	-134	940	1837	16458	2447	6956	8241	1699	4308	1273
120h after wandering(male)	-55	493	-55	-55	-55	-55	-55	-55	991	7439	-55	1294	2168	18222	2016	6422	8960	1774	4517	1054
day 6 after wandering(male)	-66	-66	503	-66	1895	-66	-66	1288	3446	3599	-66	2231	9684	18592	1709	3574	8626	2350	3331	1810
day 6 after wandering(male)	-87	-87	834	-87	1584	-87	-87	912	3936	1975	-87	1584	7107	15137	1227	2798	5775	1340	2716	2835
day 7 after wandering(male)	-293	-293	-293	-293	-293	-293	-293	1219	-293	-293	-293	-293	14991	3902	11484	2697	8209	1665	3219	1624
day 7 after wandering(male)	-87	-87	-87	-87	-87	-87	-87	893	-87	-87	-87	-87	16252	2069	7388	1924	6950	1030	1920	1391
day 8 after wandering(male)	-346	-346	-346	-346	5121	-346	-346	-346	-346	-346	-346	-346	2049	3864	1513	3964	5194	2894	2794	1696
day 8 after wandering(male)	-146	-146	-146	-146	5363	-146	-146	-146	-146	-146	-146	-146	2870	2503	1629	4569	6128	4841	3111	1891
day 9 after wandering(male)	-283	-283	-283	-283	13802	2791	2130	-283	-283	-283	-283	-283	4774	-283	1447	3445	8041	4760	3986	-283
day 9 after wandering(male)	-117	-117	-117	-117	11010	2178	1821	-117	-117	-117	-117	-117	6271	-117	1672	3299	8963	5853	3900	-117
day 10 after wandering(male)	-88	475	823	-88	30559	14489	1619	1466	-88	-88	1377	1420	963	6148	-88	982	4207	13966	495	3025
day 10 after wandering(male)	-83	542	1040	-83	42519	827	8129	2056	-83	-83	831	1887	1058	5671	-83	1366	4692	5866	4152	4426
adult(male)	1073	-146	-146	-146	15290	15540	7021	-146	-146	-146	-146	2004	1608	4500	4671	2902	6364	17490	9176	1470
adult(male)	1041	-109	-109	-109	17648	16977	8344	-109	-109	-109	-109	2515	1780	3049	3802	4243	9210	19049	8985	1628

Developmental stage	BmSPI80	BmSPI79	BmSPI70	BmSPI57	BmSPI55	BmSPI65	BmSPI66	BmSPI38	BmSPI36	BmSPI45	BmSPI49	BmSPI37	BmSPI47	BmSPI39	BmSPI46	BmSPI48	BmSPI3	BmSPI17	BmSPI24	BmSPI16/18/22
day 3 of fifth instar(mix)	2788	-88.1667	7540.333	4963.5	9259.833	2834.833	10676.67	37563	21284	11939.17	9975.833	689.3333	-88.1667	-88.1667	-88.1667	3050	-88.1667	-88.1667	-88.1667	20325.33
day 4 of fifth instar(male)	1947	-56	5516	5806	4654	1638	12811	37194	18412	37265	5000	1970	1220	2217	-56	1250	1311	-56	1862	33235
day 4 of fifth instar(male)	1275	-73	8977	3841	5474	2227	13896	47648	29845	47627	3343	1765	1108	1622	-73	874	2008	-73	2772	36382
day 5 of fifth instar(male)	-112	-112	7228	3250	2762	1936	12800	44140	17439	62765	5792	2508	6668	3751	1938	1536	-112	1015	2718	32708
day 5 of fifth instar(male)	-92	-92	6126	4937	2911	1834	16953	34738	14088	60424	5476	1760	6203	4383	2259	885	-92	3740	1954	22500
day 6 of fifth instar(male)	-109	-109	8538	2725	2651	3958	19172	21715	10682	71567	2550	3851	7692	12232	1624	-109	-109	3229	6112	9765
day 6 of fifth instar(male)	-135	-135	6362	2940	2463	3781	15776	14094	8437	88333	2725	4045	10984	14614	1844	-135	-135	2436	6671	7926
day 7 of fifth instar(male)	-157	-157	2611	1781	1810	1347	8835	38455	7811	50844	1263	2484	7530	6225	1715	-157	-157	1404	3644	11803
day 7 of fifth instar(male)	-87	-87	2343	2046	2089	1233	7881	30721	5023	56708	1263	1818	5870	9117	2369	-87	-87	1798	3536	11368
start of wandering(male)	1754	-116	1742	2188	4607	1511	7644	42286	7694	39350	4018	1927	5255	5238	-116	1204	-116	2370	2123	18023
start of wandering(male)	1900	-72	1426	3199	4679	1813	5163	33077	5457	41391	4245	1735	5145	4982	-72	1115	-72	3721	1639	19843
12h after wandering(male)	1035	-64	-64	1208	1310	816	14267	3492	1219	3689	1291	-64	4221	708	557	-64	-64	-64	872	-64

12h after wandering(male)	1530	-73	-73	790	1617	1451	12086	3784	2016	3433	942	-73	2834	823	413	-73	-73	-73	817	-73
24h after wandering(male)	1274	2932	673	3554	4607	-37	18491	2678	1529	5606	1188	-37	12582	951	639	-37	-37	-37	585	-37
24h after wandering(male)	946	3628	626	3441	4034	-76	16648	3433	1574	4480	959	-76	9538	584	351	-76	-76	-76	583	-76
36h after wandering(male)	1662	4608	671	5045	6563	547	13816	3180	1488	2915	882	-65	6964	718	-65	-65	-65	-65	-65	-65
36h after wandering(male)	1789	5476	802	5046	7177	771	10599	3108	1283	2667	894	-105	8900	802	-105	-105	-105	-105	-105	-105
48h after wandering(male)	1335	3363	-52	7058	5779	500	13904	3465	2354	1339	954	897	828	2081	-52	-52	-52	-52	-52	-52
48h after wandering(male)	1245	3576	-98	6432	6418	583	19205	3957	2835	1777	991	855	855	2004	-98	-98	-98	-98	-98	-98
60h after wandering(male)	1074	4936	1369	12365	11225	3111	11499	4223	1803	1700	855	487	-66	941	-66	-66	-66	-66	-66	-66
60h after wandering(male)	505	3829	1645	8221	12592	3782	14602	3916	1893	1487	1028	702	-54	928	-54	-54	-54	-54	-54	-54
72h after wandering(male)	1212	1712	1694	5558	3301	-198	28236	6066	3506	-198	-198	-198	-198	-198	-198	-198	-198	-198	-198	-198
72h after wandering(male)	1424	1496	1624	7782	2940	-126	25751	4127	2724	-126	-126	-126	-126	-126	-126	-126	-126	-126	-126	-126
96h after wandering(male)	735	1329	1230	10963	3676	-91	22528	3283	2679	1398	-91	-91	-91	-91	-91	-91	-91	-91	-91	-91
96h after wandering(male)	1001	1316	1191	14033	4299	-79	26067	3119	2711	1135	-79	-79	-79	-79	-79	-79	-79	-79	-79	-79
120h after wandering(male)	525	2252	3150	12612	-134	-134	17473	4235	2718	1263	-134	-134	-134	-134	-134	-134	-134	-134	-134	-134
120h after wandering(male)	846	2519	3068	15775	-55	-55	20950	2417	2170	1091	-55	-55	-55	-55	-55	-55	-55	-55	-55	-55
day 6 after wandering(male)	1045	2883	1417	15885	19589	1143	6204	3141	1286	1852	-66	-66	-66	-66	-66	-66	-66	-66	-66	-66
day 6 after wandering(male)	599	1492	1108	10287	16801	1176	5174	4156	1671	2259	-87	-87	-87	-87	-87	-87	-87	-87	-87	-87
day 7 after wandering(male)	-293	7209	2891	10379	4386	2706	22184	3175	4802	-293	-293	-293	-293	-293	-293	-293	-293	-293	-293	-293
day 7 after wandering(male)	-87	5336	2007	8711	2860	2887	12582	3405	2860	-87	-87	-87	-87	-87	-87	-87	-87	-87	-87	-87
day 8 after wandering(male)	-346	-346	-346	12496	13902	-346	4037	5284	3275	2707	-346	-346	-346	-346	-346	-346	-346	-346	-346	-346
day 8 after wandering(male)	-146	-146	-146	14516	15404	-146	4672	5317	3191	2708	-146	-146	-146	-146	-146	-146	-146	-146	-146	-146
day 9 after wandering(male)	1362	2074	2907	9764	15743	-283	7911	4247	5372	2009	-283	-283	-283	-283	-283	-283	-283	-283	-283	-283
day 9 after wandering(male)	1406	1889	2302	13352	16098	-117	9404	4668	2859	2050	-117	-117	-117	-117	-117	-117	-117	-117	-117	-117
day 10 after wandering(male)	4467	391	4667	28215	675	2290	7014	528	2363	1618	3707	556	-88	-88	-88	1679	-88	-88	-88	1009
day 10 after wandering(male)	1612	521	9166	13998	21012	2137	7184	4603	3199	1527	1984	588	-83	-83	-83	1340	-83	-83	-83	719
adult(male)	7769	-146	6631	95483	15774	3974	7515	8938	2103	1749	4518	858	-146	-146	-146	828	-146	-146	-146	-146
adult(male)	9497	-109	5756	71369	12808	5504	5143	10384	2123	1893	5203	869	-109	-109	-109	1259	-109	-109	-109	-109

Developmental stage	BmSPI61	BmSPI62	BmSPI76	BmSPI72	BmSPI71	BmSPI52	BmSPI53	BmSPI56	BmSPI68	BmSPI27	BmSPI15	BmSPI25	BmSPI6	BmSPI10	BmSPI12	BmSPI9	BmSPI5	BmSPI11	BmSPI4	BmSPI2
day 3 of fifth instar(mix)	-88.166667	-88.1667	-88.1667	-88.1667	-88.1667	-88.1667	-88.1667	-88.1667	-88.1667	-88.1667	-88.1667	-88.1667	-88.1667	330.6667	2299.833	24849	10002.5	8567.5	3744.333	4069.667
day 4 of fifth instar(female)	-73	-73	-73	-73	-73	-73	-73	-73	-73	-73	472	-73	-73	942	2073	27709	3015	7038	1811	2031
day 4 of fifth instar(female)	-80	-80	-80	-80	-80	-80	-80	-80	-80	-80	747	-80	-80	1014	1762	33016	4579	5245	2774	2806
day 5 of fifth instar(female)	-113	-113	-113	-113	-113	-113	-113	-113	-113	-113	-113	-113	-113	425	1304	28648	3558	8777	2532	3324
day 5 of fifth instar(female)	-83	-83	-83	-83	-83	-83	-83	-83	-83	-83	-83	-83	-83	512	1266	26181	4023	7510	2273	3864
day 6 of fifth instar(female)	-143	-143	-143	-143	-143	-143	-143	-143	-143	-143	-143	-143	-143	2750	5327	44044	8132	5111	3979	1763
day 6 of fifth instar(female)	-128	-128	-128	-128	-128	-128	-128	-128	-128	-128	-128	-128	-128	1890	5649	39508	8078	3859	4410	2248
day 7 of fifth instar(female)	-131	-131	-131	-131	-131	-131	-131	-131	-131	-131	-131	-131	1047	1866	5101	9820	5894	3508	2090	881
day 7 of fifth instar(female)	-145	-145	-145	-145	-145	-145	-145	-145	-145	-145	-145	-145	1092	1266	2755	6678	5582	2536	2271	1708
start of wandering(female)	-66	338	-66	-66	-66	-66	-66	-66	-66	-66	-66	-66	-66	936	4219	9062	10388	5819	1900	1879
start of wandering(female)	-67	608	-67	-67	-67	-67	-67	-67	-67	-67	-67	-67	-67	1693	5254	10688	16063	7930	2186	1799
12h after wandering(female)	-104	-104	-104	-104	-104	-104	-104	-104	-104	-104	-104	473	2381	655	1051	1729	12079	5080	3452	1198
12h after wandering(female)	-76	-76	-76	-76	-76	-76	-76	-76	-76	-76	-76	592	3030	949	1782	1679	11015	6514	3387	906
24h after wandering(female)	-196	-196	-196	-196	-196	-196	-196	-196	-196	888	-196	1146	2896	-196	1846	2778	29964	10500	3991	1456
24h after wandering(female)	-42	-42	-42	-42	-42	-42	-42	-42	-42	653	-42	672	3231	-42	1915	1595	16238	7756	3831	1095
36h after wandering(female)	-161	-161	-161	-161	-161	-161	-161	-161	4770	3213	-161	-161	5931	5779	-161	2053	13792	8905	2040	1343
36h after wandering(female)	-116	-116	-116	-116	-116	-116	-116	-116	2935	3677	-116	-116	8087	3973	-116	2018	14073	8989	2119	1158
48h after wandering(female)	-127	-127	-127	-127	-127	-127	-127	-127	-127	-127	-127	-127	10445	1144	-127	2526	16029	16814	4446	2216
48h after wandering(female)	-251	-251	-251	-251	-251	-251	-251	-251	-251	-251	-251	-251	9994	1638	-251	2488	20166	18075	4061	2264
60h after wandering(female)	-61	540	-61	391	-61	-61	-61	-61	-61	644	-61	1494	1780	514	1893	3319	16178	18256	3362	2254
60h after wandering(female)	-50	556	-50	451	-50	-50	-50	-50	-50	640	-50	1393	1679	489	1701	2709	14857	17963	3502	2948
72h after wandering(female)	-183	-183	-183	-183	-183	-183	-183	-183	-183	-183	-183	999	1134	1969	1588	4014	8953	3860	2866	1080
72h after wandering(female)	-74	-74	-74	-74	-74	-74	-74	-74	-74	-74	-74	1202	1582	1906	1630	3331	9601	3759	3590	1189
96h after wandering(female)	-261	-261	-261	-261	-261	-261	-261	-261	-261	1359	-261	-261	-261	1898	-261	5342	5860	3818	2685	1216
96h after wandering(female)	-119	-119	-119	-119	-119	-119	-119	-119	-119	1594	-119	-119	-119	2036	-119	7314	6592	2587	3529	1101
120h after wandering(female)	-77	-77	-77	-77	-77	-77	-77	-77	782	5685	-77	766	1586	13874	2269	6141	6813	1357	2554	858
120h after wandering(female)	-102	-102	-102	-102	-102	-102	-102	-102	786	5047	-102	797	1513	15436	1650	4533	8221	1615	3078	1134

day 6 after wandering(female)	-67	-67	-67	-67	1146	480	-67	-67	-67	1609	-67	1436	1997	3727	1961	2377	6870	1612	2403	1478
day 6 after wandering(female)	-60	-60	-60	-60	1995	651	-60	-60	-60	1415	-60	1104	2241	3058	1727	1617	4955	1799	2282	1158
day 7 after wandering(female)	-227	-227	-227	-227	-227	-227	-227	-227	2240	1226	-227	-227	2918	16860	1753	2065	3549	-227	2234	1135
day 7 after wandering(female)	-84	-84	-84	-84	-84	-84	-84	-84	1189	796	-84	-84	2720	10741	1186	1339	3042	-84	2196	1549
day 8 after wandering(female)	-97	-97	-97	-97	-97	-97	-97	-97	-97	-97	-97	516	1106	-97	1159	1824	3329	1447	1873	1260
day 8 after wandering(female)	-90	-90	-90	-90	-90	-90	-90	-90	-90	-90	-90	610	1505	-90	1252	2006	3432	1660	1680	1602
day 9 after wandering(female)	-141	-141	-141	-141	-141	-141	-141	805	-141	-141	-141	-141	-141	1643	-141	3001	4626	2016	2390	1464
day 9 after wandering(female)	-96	-96	-96	-96	-96	-96	-96	848	-96	-96	-96	-96	-96	1404	-96	1977	5359	1466	2114	2165
day 10 after wandering(female)	-87	-87	544	-87	-87	-87	-87	997	-87	-87	570	967	279	3882	-87	690	2927	1593	1100	3024
day 10 after wandering(female)	-100	-100	762	-100	-100	-100	-100	1998	-100	-100	681	1149	560	1729	-100	1371	3211	1707	1931	5342
adult(female)	-88	1162	866	-88	-88	-88	-88	-88	-88	-88	-88	2874	3002	2817	5838	2578	6495	9025	4673	2792
adult(female)	-155	865	840	-155	-155	-155	-155	-155	-155	-155	-155	1950	2410	1922	4371	3090	5959	9501	4152	3352

Developmental stage	BmSPI80	BmSPI79	BmSPI70	BmSPI57	BmSPI55	BmSPI65	BmSPI66	BmSPI38	BmSPI36	BmSPI45	BmSPI49	BmSPI37	BmSPI47	BmSPI39	BmSPI46	BmSPI48	BmSPI3	BmSPI17	BmSPI24	BmSPI16/18/22
day 3 of fifth instar(mix)	2788	-88.1667	7540.333	4963.5	9259.833	2834.833	10676.67	37563	21284	11939.17	9975.833	689.3333	-88.1667	-88.1667	-88.1667	3050	-88.1667	-88.1667	-88.1667	20325.33
day 4 of fifth instar(female)	799	-73	3184	3194	2964	1152	13634	48245	15190	48404	3022	1803	2972	1383	1229	924	1887	2563	2482	35258
day 4 of fifth instar(female)	1118	-80	3370	3035	4633	2644	10835	52556	16985	52519	2582	2150	2846	1511	1223	1098	3792	3176	3347	36849
day 5 of fifth instar(female)	885	-113	3418	3066	3073	1311	11992	72232	21549	62246	4876	2115	-113	2854	1499	1069	-113	879	-113	25309
day 5 of fifth instar(female)	1644	-83	2569	4326	3510	1094	7372	42905	16188	44021	4152	1517	-83	2655	1420	774	-83	2125	-83	15738
day 6 of fifth instar(female)	1032	-143	6752	1694	4124	3628	29495	93593	19010	63946	2269	6077	5731	6010	1241	-143	-143	-143	5201	29336
day 6 of fifth instar(female)	1518	-128	5801	2150	4155	4563	26723	82193	15232	51341	1948	4661	6227	7085	1053	-128	-128	-128	6136	26175
day 7 of fifth instar(female)	-131	-131	1272	1340	2163	5514	-131	17539	4475	16286	-131	3707	5097	10044	-131	-131	-131	-131	2230	2187
day 7 of fifth instar(female)	-145	-145	1009	1786	2253	3502	-145	7094	4068	10893	-145	2999	4394	12883	-145	-145	-145	-145	2413	1854
start of wandering(female)	1135	532	839	2053	3784	1435	3598	19534	3952	27157	2032	2084	2880	14727	453	515	-66	1856	1339	1632
start of wandering(female)	1403	764	1167	2883	3632	1918	6306	16188	4008	33295	2537	1645	2863	9789	621	608	-67	2337	1780	1837
12h after wandering(female)	888	1445	529	1198	2090	703	12931	2954	1813	-104	1589	469	5565	1424	571	-104	-104	438	531	-104
12h after wandering(female)	1174	1610	453	1809	1766	637	14551	2567	1753	-76	2087	375	5382	1380	727	-76	-76	1252	785	-76
24h after wandering(female)	1946	7885	-196	6602	6832	967	12390	2732	1378	7706	2783	-196	19255	-196	-196	-196	-196	-196	-196	-196
24h after wandering(female)	1311	4449	-42	4869	5355	712	25602	1833	1073	6805	1569	-42	11963	-42	-42	-42	-42	-42	-42	-42
36h after wandering(female)	1424	4961	-161	4556	10722	1013	10192	3127	1361	2100	1152	-161	6979	-161	-161	-161	-161	-161	-161	-161
36h after wandering(female)	2173	5177	-116	5944	12572	979	8617	2108	843	2276	1237	-116	8330	-116	-116	-116	-116	-116	-116	-116
48h after wandering(female)	1150	2839	-127	10075	7261	-127	21352	4293	3405	1365	-127	-127	-127	-127	-127	-127	-127	-127	-127	-127
48h after wandering(female)	1347	4899	-251	10755	12580	-251	31012	5348	2991	1249	-251	-251	-251	-251	-251	-251	-251	-251	-251	-251
60h after wandering(female)	1875	4862	1471	19829	14507	3229	20208	4042	1675	1783	515	-61	-61	-61	-61	-61	-61	-61	-61	-61
60h after wandering(female)	1492	4062	1271	17965	15769	3414	15935	3598	1603	1528	785	-50	-50	-50	-50	-50	-50	-50	-50	-50
72h after wandering(female)	1081	2020	-183	8757	3749	-183	27893	5422	1815	981	-183	-183	-183	-183	-183	-183	-183	-183	-183	-183
72h after wandering(female)	1615	1840	-74	11284	3461	-74	24584	4516	1714	1059	-74	-74	-74	-74	-74	-74	-74	-74	-74	-74
96h after wandering(female)	-261	1422	-261	14843	4443	-261	25337	3420	3067	-261	-261	-261	-261	-261	-261	-261	-261	-261	-261	-261
96h after wandering(female)	-119	1529	-119	12927	5817	-119	16374	2966	2910	-119	-119	-119	-119	-119	-119	-119	-119	-119	-119	-119
120h after wandering(female)	689	1949	1250	13025	6039	-77	23579	2954	2378	783	-77	-77	-77	-77	-77	-77	-77	-77	-77	-77
120h after wandering(female)	677	2019	1304	12208	5595	-102	17910	2171	2194	806	-102	-102	-102	-102	-102	-102	-102	-102	-102	-102
day 6 after wandering(female)	1188	8757	1361	14689	20428	813	4355	2314	1224	1056	-67	-67	-67	-67	-67	-67	403	-67	-67	-67
day 6 after wandering(female)	1465	7039	1029	16098	16774	881	3599	2902	1106	1045	-60	-60	-60	-60	-60	-60	633	-60	-60	-60
day 7 after wandering(female)	-227	1137	-227	4985	4835	-227	4974	3380	3244	-227	-227	-227	-227	-227	-227	-227	-227	-227	-227	-227
day 7 after wandering(female)	-84	989	-84	6279	3427	-84	3650	2608	2669	-84	-84	-84	-84	-84	-84	-84	-84	-84	-84	-84
day 8 after wandering(female)	794	-97	-97	7455	6634	-97	3373	3340	2170	1063	-97	1396	-97	-97	-97	-97	-97	-97	-97	553
day 8 after wandering(female)	833	-90	-90	9270	6041	-90	4188	3160	2709	1384	-90	1349	-90	-90	-90	-90	-90	-90	-90	712
day 9 after wandering(female)	1575	-141	2369	11038	6627	-141	10022	5393	4145	1458	861	4175	-141	-141	-141	-141	-141	-141	-141	-141
day 9 after wandering(female)	1064	-96	2876	10913	4983	-96	10281	3848	4329	1211	822	4919	-96	-96	-96	-96	-96	-96	-96	-96
day 10 after wandering(female)	1591	-87	3613	10311	3609	1789	7173	3409	3255	938	1099	629	-87	-87	617	636	-87	-87	-87	1218
day 10 after wandering(female)	643	-100	3892	8906	3154	1342	7704	3148	3426	2318	1414	736	-100	-100	632	913	-100	-100	-100	1673
adult(female)	6981	-88	6052	20246	9544	3108	6766	6485	1939	2433	4774	-88	1929	-88	1260	1037	-88	-88	-88	-88
adult(female)	4135	-155	5677	17146	9283	2241	7873	7897	2256	2547	4591	-155	1556	-155	1006	982	-155	-155	-155	-155
