# Supplementary material for: Genome-Wide Identification and Immune Response Analysis of Serine Protease Inhibitor Genes in the Silkworm, Bombyx mori
Source: PLoS One. 2012 Feb 13;7(2):e31168. doi: 10.1371/journal.pone.0031168 (PMC3278429; doi:10.1371/journal.pone.0031168)
Supplement: Table S4 — Ratios and annotations of microorganism induced SPI genes. (PDF) [file pone.0031168.s008.pdf]

Supporting Information Table 4

Ratios and annotations of microorganism induced SPI genes

Microorganism	BmSPI80	BmSPI11	BmSPI6	BmSPI24	BmSPI28	BmSPI17	BmSPI16/18/22	BmSPI37	BmSPI46	BmSPI48	BmSPI39	BmSPI45	BmSPI47	BmSPI49	BmSPI55	BmSPI70	BmSPI62
E.coli3h	1.5957	1.22885	0.741	0.0843	-	0.2996	1.3312	0.6747	0.6007	0.5312	0.61115	0.47955	-	0.58885	1.7743	1.1608	1.0423
E.coli6h	1.2139	1.01215	0.9769	0.788	2.07225	0.8223	0.5754	1.0365	1.1358	0.89375	1.10165	0.8062	-	0.8511	1.231	0.9534	-
E.coli12h	0.8296	1.3425	0.7373	0.792	-	0.7918	1.0798	0.82335	0.84355	0.7228	1.0531	1.19375	-	0.7683	0.9236	0.9723	-
E.coli24h	1.0928	2.6122	2.22585	1.7589	4.70535	1.48255	1.1646	3.80725	2.5068	1.8943	2.03015	2.98865	3.7196	1.55675	0.4378	0.5762	0.5781
B.bombysepticus3h	1.4816	2.07915	1.24175	0.08985	-	0.2487	1.00195	0.7973	0.22395	0.6543	0.3566	0.4254	-	0.76525	1.655	1.2921	-
B.bombysepticus6h	0.8757	1.25435	0.91295	0.66275	1.47415	0.32995	0.94985	1.0846	0.6916	0.472	0.7186	0.85295	-	0.4989	1.1852	0.8929	0.9671
B.bombysepticus12h	0.8958	1.11155	0.7001	0.32335	0.47005	0.2859	0.8946	0.78215	0.4914	0.62445	0.5184	0.61605	-	0.68455	1.2883	1.1423	-
B.bombysepticus24h	2.5562	1.21205	0.66095	0.8171	0.73375	0.4831	0.671	2.15985	2.1383	1.01385	0.55215	0.87245	-	0.8937	0.47365	0.4073	0.2865
B.bassiana6h	0.7977	0.6378	0.3864	1.17395	1.05155	0.35835	0.80915	1.06705	1.0567	0.84525	1.1341	0.7107	-	0.89605	1.12915	1.0487	1.2124
B.bassiana12h	0.9827	1.11595	1.15515	1.489	1.52455	1.77335	0.80695	0.86405	0.9032	1.102	1.6744	0.9684	-	1.0815	0.96305	1.1426	1.3252
B.bassiana24h	2.5650	2.00885	0.68345	0.9273	0.63715	1.0023	0.3318	1.8088	1.4523	0.7255	0.76515	1.1203	-	0.61545	0.56625	0.3601	0.3254
B.bassiana48h	3.0628	1.50745	0.8897	0.50215	0.31995	1.23255	0.84385	0.5988	1.1691	2.1426	0.6107	1.3004	0.2917	-	0.8563	1.0119	0.6788
BmNPV3h	1.1222	1.3276	0.9718	0.13545	-	0.15905	0.94445	0.75945	0.31865	0.51005	0.40175	0.52485	-	0.54575	1.55555	0.9592	-
BmNPV6h	0.4417	0.9952	2.633	0.1956	-	0.0924	1.2311	0.42685	0.37935	0.89835	0.5692	0.47115	-	1.11725	2.74895	2.6761	1.8664
BmNPV12h	0.8637	0.95535	0.51125	0.48665	0.8276	0.4863	1.09265	0.88955	0.57725	0.5057	0.6989	0.55765	-	0.4849	1.27705	1.1011	-
BmNPV24h	1.4067	0.9118	0.28675	0.68125	0.8624	0.7517	0.8804	1.4916	0.85235	0.5578	0.898	0.9069	-	0.54535	0.69115	0.8662	0.7264
