# Supplementary material for: Genome-Wide Identification and Immune Response Analysis of Serine Protease Inhibitor Genes in the Silkworm, Bombyx mori
Source: PLoS One. 2012 Feb 13;7(2):e31168. doi: 10.1371/journal.pone.0031168 (PMC3278429; doi:10.1371/journal.pone.0031168)
Supplement: Table S5 — Primer sequences, sizes of PCR production and melting temperature for semi-quantitative RT-PCR. (PDF) [file pone.0031168.s009.pdf]

Supporting Information Table 5

Primer sequences, sizes of PCR production and melting temperature for semi-quantitative RT-PCR

Gene	(5'-3')	Primer	Size(bp)	Melting temperature(℃)
BmSPI16	Forward	GGCGTCAACCAGATTGCCT	876	56
	Reverse	TCAGTGCGTGATAAGGCCAACG		
BmSPI17	Forward	CGGCGAAGGTGAAGTATACTCG	451	56
	Reverse	CATCGACAACTACGCCTATTATGG		
BmSPI18	Forward	AACGTTAACCAGGCCGTCA	876	56
	Reverse	TCAGTTTGTAATAAGGCCAATG		
BmSPI22	Forward	TACGTCAACCAGGCCGTCA	876	56
	Reverse	TCAGGTCGTAATAATGCCAATG		
BmSPI36	Forward	GAGCTTGTTCGAGAAATCGT	180	56
	Reverse	AACATTCGGCAACGGGCA		
BmSPI37	Forward	AGCTGGTTCGAAAACCCGT	209	56
	Reverse	TCACTTGAAATAGCCACCG		
BmSPI38	Forward	ATCGTGTTCCTGATTGTCG	207	56
	Reverse	CAATCAGAAATGGGCACAC		
BmSPI45	Forward	GCTATAATCGCTCAGTGCC	387	56
	Reverse	CTTCATTCGGTCTGGTGC		
BmSPI47	Forward	CGCTGTTGTTGGATGTGG	539	56
	Reverse	GTGGGCACTGGTTTGGTT		
BmSPI49	Forward	CGTCAACATCATCGTCCTC	573	56
	Reverse	GTGCCATTGTCAGCCCTA		
