# Supplementary material for: Genome-Wide Identification and Immune Response Analysis of Serine Protease Inhibitor Genes in the Silkworm, Bombyx mori
Source: PLoS One. 2012 Feb 13;7(2):e31168. doi: 10.1371/journal.pone.0031168 (PMC3278429; doi:10.1371/journal.pone.0031168)
Supplement: Table S6 — Primer sequences and sizes of PCR production for qRT-PCR. (PDF) [file pone.0031168.s010.pdf]

Supporting Information Table 6

Primer sequences and sizes of PCR production for qRT-PCR

Gene	(5'-3')	Primer	Size(bp)
BmSPI6	Forward	GGTTACTTCGTCCATTTCTTC	148
	Reverse	GGTTTAGTTCGCTTAGGATTGT	
BmSPI11	Forward	TGTCTTGATACACGAGCACTG	103
	Reverse	CTGGCGATACCATCACATTG	
BmSPI37	Forward	ACGGCTTTCATCGCAACCT	83
	Reverse	GAACATTCAGCAACAGGCAC	
BmSPI39	Forward	GACTGTAACAACTGATTCCGACTT	178
	Reverse	CGGCAATTTCATACTATAACCC	
BmSPI46	Forward	TAGATGAACGCACGATAAGG	229
	Reverse	GACGGAGCACAAAGCACTAA	
BmSPI48	Forward	TTGTCGGTTCGAGAAGTGTTC	156
	Reverse	AGAGGTCAGCGTCACAATCAC	
