# Supplementary material for: Genome-Wide Identification and Immune Response Analysis of Serine Protease Inhibitor Genes in the Silkworm, Bombyx mori
Source: PLoS One. 2012 Feb 13;7(2):e31168. doi: 10.1371/journal.pone.0031168 (PMC3278429; doi:10.1371/journal.pone.0031168)
Supplement: Figure S4 — The induced expression analyses of silkworm SPI genes by quantitative real-time RT-PCR. We chose the time points of infecting 6 h and 24 h to do the expression analysis. The expression of SPI in the control sample was set to 1. The abbreviations are used, the E. coli infected sample (A), the B. bombyseptieus infected sample (B) and the B. bassiana infected sample (C). Each expressive assay was replicated three times. The Student's t-test was used to evaluate statistical significance (* P<0.05, ** P<0. 01). (PDF) [file pone.0031168.s004.pdf]

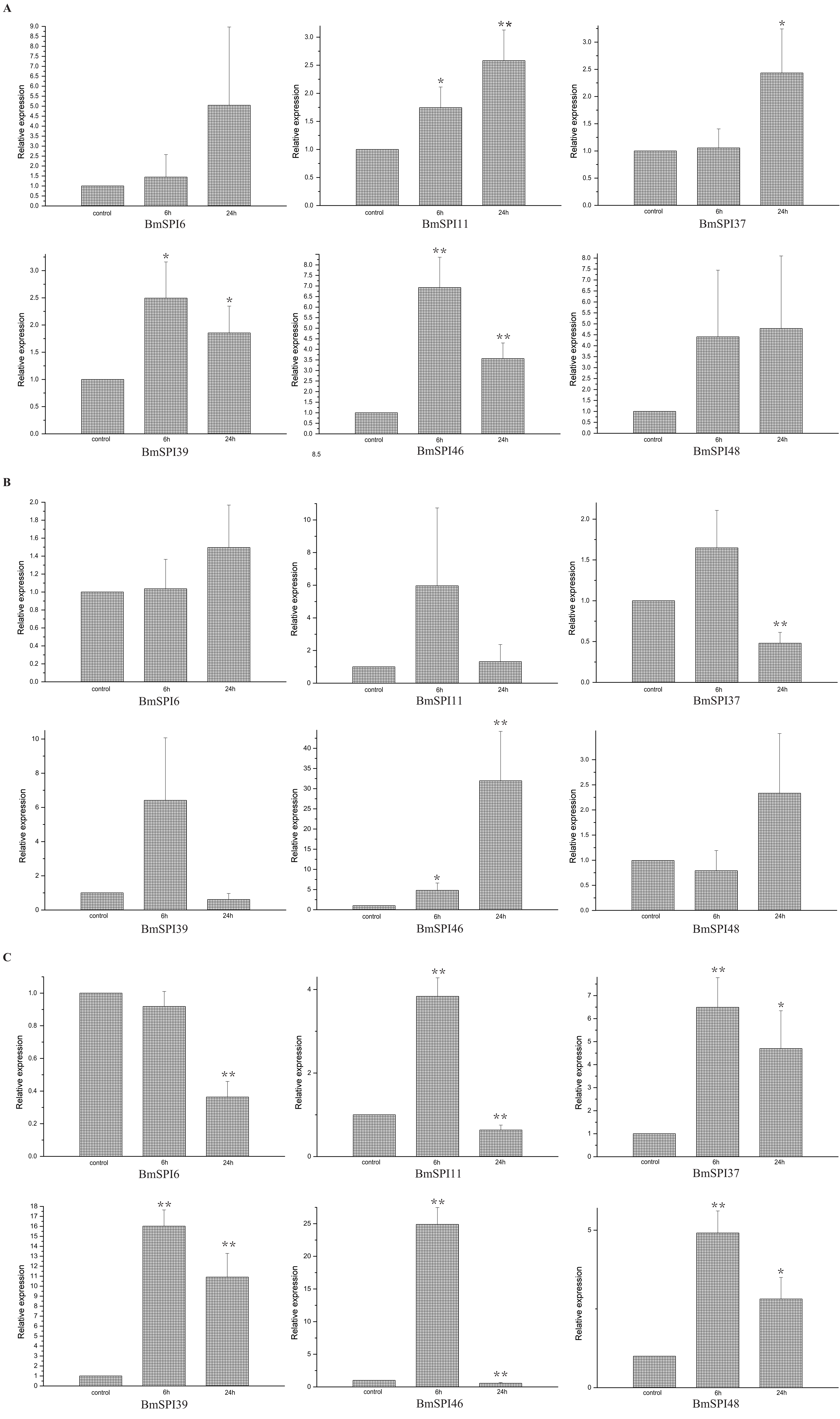
A
Relative expression
control
6h
24h
BmSPI6
BmSPI11
BmSPI37
BmSPI39
BmSPI46
8.5
BmSPI48
B
BmSPI6
BmSPI11
BmSPI37
BmSPI39
BmSPI46
BmSPI48
C
BmSPI6
BmSPI11
BmSPI37
BmSPI39
BmSPI46
BmSPI48
